# Is Blood Thicker Than Water? The Influence of Kin Within a Bull Shark Social Network

**DOI:** 10.1002/ece3.74050

**Published:** 2026-08-05

**Authors:** Natasha D. Marosi, Darren P. Croft, David M. P. Jacoby, Samuel Ellis, Andrew M. Griffiths

**Affiliations:** ^1^ Centre for Research in Animal Behaviour, College of Health and Life Sciences University of Exeter Exeter UK; ^2^ Fiji Shark Lab Pacific Harbour Deuba Fiji; ^3^ Lancaster Environment Centre Lancaster University Lancaster UK; ^4^ Department of Biosciences University of Exeter Exeter UK

**Keywords:** elasmobranch, genotyping, kinship, relatedness, single nucleotide polymorphisms, social network

## Abstract

The influence of kinship on social structure (what we novelly refer to as “kinfluence”) has been studied extensively within terrestrial taxa yet remains poorly understood in marine systems. While kin‐based associations have been explored in teleost fish and cetaceans, few have investigated this within elasmobranchs, and none have combined fine‐scale social behavior with both genomic relatedness and categorical kinship inference. This study utilized the Shark Reef Marine Reserve in Fiji, a site where a population of adult bull sharks ( *Carcharhinus leucas* ) aggregate in response to provisioning, and socially interact, to examine the role of genetic relatedness and categorical kinship in shaping social behavior. We integrated DArTseq SNP genotyping (3919 SNPs across 129 individuals) with behavioral data on active social preferences (join, lead, follow, and parallel swim events) collected over 473 observation dives conducted across 6 years. Social network analyses revealed a structured network driven by active preferences with genomic relatedness exerting a small yet significant influence on social behavior at both dyadic and community levels, particularly within male—male dyads. Pedigree reconstruction identified full‐ and half‐sibling dyads, allowing direct examination of kin‐category effects. At the community level, analysis utilizing a weighted kinship matrix revealed associations between full and half‐sibling dyads were slightly stronger than between non‐kin dyads, whereas no detectable relationship was uncovered for social interactions at the dyadic level. Our mixed findings may reflect differences in the datasets or be partially explained by sibship reconstruction omitting third order relationships (first cousin avuncular) in defining kin categories. Our study provides rare empirical support for kin‐selection in a large marine predator, underscoring the need to further investigate the role of kin in shaping social systems within elasmobranchs.

## Graphical abstract

This study investigates how kinship (“kinfluence”) shapes social structure in an aggregation of adult bull sharks in Fiji by integrating genomic relatedness, pedigree reconstruction, and detailed behavioral data. It reveals that while genetic relatedness has a small but significant influence on social behavior, especially among males, kin‐category effects are weak and inconsistent, with only slight increases in association among close kin. Overall, the findings provide rare empirical support for kin‐based processes in a large marine predator, while highlighting the complexity of social dynamics in elasmobranchs.

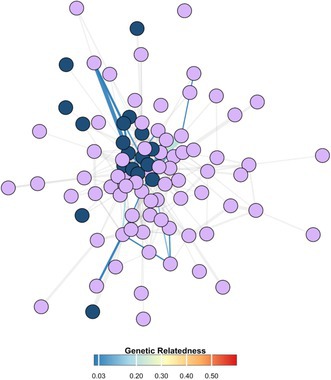

## 1 Introduction

Social grouping and social behavior are adaptive strategies exhibited by a wide range of animal species, and these strategies can confer several fitness benefits (Krause and Ruxton 2002). Across different species and within populations, social structures and sociality can vary considerably, having fundamental consequences for ecology and evolution (Krause and Ruxton 2002; Kurvers et al. 2014; Snijders et al. 2017; Snyder‐Mackler et al. 2020). Identifying the drivers which underpin animal social groups and their evolution gives us access to invaluable tools to bolster our understanding of population dynamics and inform conservation strategy (Kurvers et al. 2014; Snijders et al. 2017; Snyder‐Mackler et al. 2020). Social grouping and social behavior can confer benefits to individuals which include increasing foraging success and access to potential mates, providing opportunities for social learning and information transfer, but also anti‐predator defense mechanisms, with possibilities for the formation of cooperative bonds (Beck et al. 2021; Berdahl et al. 2018; Croft et al. 2006; Farine and Sheldon 2015; Gokcekus et al. 2021; Krause and Ruxton 2002; Lehtonen and Jaatinen 2016; Ripperger et al. 2019). These benefits however, must be traded off against the costs attributed to group membership, which include parasite and disease transmission, competition and conflict over finite resources and mating opportunities (Krause et al. 2007; Wilkinson et al. 2016). Where this balance of trade offs results in the formation of social groups, they may take on many forms: societies may be simple or complex in structure, short lived or long term, stable or characterized by fission‐fusion dynamics (Croft et al. 2008; M. J. Silk 2023). Within these societies, individuals may also exhibit preferences to group or associate with individuals that share similar phenotypic traits such as sex, size, personality, or that are genetically related (Croft et al. 2008; Farine 2014).

The kinship structure of groups, that is, how individual members are related to one another is a key component driving both within group cooperation and the evolution of group living and interaction (Pereira et al. 2023). Kin structured social groups can provide enhanced benefits to members through inclusive fitness benefits—through for example, increasing the fitness of group members by sharing information regarding resources, cooperative foraging, group predator inspection and evasion, providing social tolerance and coalitionary support (Hamilton 1964a, 1964b; Hepper 1986; Milinski 1987; Smith 2014). However, inclusive fitness benefits provided by associating with kin are not fixed and result from the complex interplay between environmental context, resource availability and the individuals themselves (Schneider and Bilde 2008; Vitt et al. 2020). Studying dyadic kinship relationships can aid our understanding of the evolution of cooperation, the reduction of conflict and aggression and inbreeding avoidance (Hamilton 1963, 1964a, 1964b; Pusey 1987). Examining kinship structure at the level of the community can inform on the nature of collective behaviors, on cooperation among kin and non‐kin dyads within groups and the amelioration of conflict (Croft et al. 2021; Grout et al. 2024; Rueger et al. 2021). Investigating kin composition at the level of the population can facilitate our understanding of dispersal patterns, genetic diversity and connectivity (Carrier et al. 2004; Hamilton 1964a, 1964b).

Understanding the mechanisms shaping social structure has advanced in terrestrial systems, whereas comparable research in marine environments has faced logistical constraints, limited accessibility, and challenges in identifying individuals (Jacoby et al. 2022). Early marine studies focused largely on teleost fish (Arnold 2000; Behrmann‐Godel et al. 2006; Griffiths and Ward 2011; Hain and Neff 2009; Makowicz et al. 2016; Thünken et al. 2016). Subsequent work on cetaceans demonstrated that social associations are often correlated with genetic relatedness in species such as bottlenose ( *Tursiops truncatus* ) and common ( *Delphinus delphis* ) dolphins, (Diaz‐Aguirre et al. 2019; Frère et al. 2010; Möller et al. 2006; Parsons et al. 2003; J. B. Silk 2002; Wiszniewski et al. 2010). However, investigations of kinship and social structure in elasmobranchs remain limited and have yielded mixed results. Kin‐structured associations have been reported in juvenile bluntnose sixgill sharks ( *Hexanchus griseus* ) and juvenile lemon sharks ( *Negaprion brevirostris* ) in nursery environments (Andrews et al. 2009; Guttridge et al. 2011; Larson et al. 2011). In contrast, a study on juvenile small spotted catsharks ( *Scyliorhinus canicula* ) conducted under controlled laboratory conditions found no influence of maternal relatedness on social structure (Jacoby et al. 2012b). Similarly, studies on adult elasmobranchs, including spotted eagle rays ( *Aetobatus narinari* ) and blacktip reef sharks (*Carcharchinus melantopterus*), have generally reported a lack of kin‐structured sociality, likely reflective of the ecological context, the absence of parental care in this taxonomic group, and fission‐fusion dynamics (Mourier and Planes 2021; Newby et al. 2014). However, most elasmobranch behavioral studies infer social structure from spatial co‐occurrence rather than direct measures of behavioral interactions, leaving a critical gap in understanding how kinship influences fine‐scale social decisions in sharks.

Our study species, the bull shark ( *Carcharhinus leucas* ), is circumglobally distributed in inshore and coastal areas of tropical and warm temperate waters (Compagno et al. 2005), and is euryhaline, reported in marine, brackish, and freshwater environments (Dwyer et al. 2020). It is characterized by a conservative life history strategy: slow growth rate, late‐stage sexual maturity, low fecundity, and high levels of maternal investment (Dulvy et al. 2014; Field et al. 2009) and is particularly vulnerable to local extinction within Fiji due to excessive harvesting at early life stages via illegal deployment of gill nets within riverine and estuary systems. Genetic research has also shown that Fiji's *C. leucas* population is isolated (Devloo‐Delva et al. 2023; Glaus et al. 2020). Bull sharks show viviparity, meaning there are even opportunities before birth for the formation of social relationships. In the context of Fiji, pregnant females display reproductive philopatry within riverine systems, where they can give birth biennially to between 1 and 13 pups (but usually 1–6) (Bass et al. 1973; Brunnschweiler et al. 2024; Compagno et al. 2005; Ebert et al. 2013; Glaus 2019). Genetic sampling of pups from riverine systems within Fiji's main island of Viti Levu has provided evidence of multiple paternity in litters (Glaus 2019). Shortly after birth, female bull sharks return to the ocean, leaving their offspring to inhabit freshwater and estuarine areas for several years (Brunnschweiler et al. 2024; Cardeñosa et al. 2017; Glaus et al. 2019b). Neonates, young‐of‐the‐year (YOY), and juveniles have all been repeatedly identified in riverine habitats through fisheries independent surveys, indicating prolonged residency during early life stages (Cardeñosa et al. 2017; Glaus et al. 2019b). Shared home ranges within these restricted environments may facilitate repeated encounters and the emergence of social behaviors such as group foraging, predator inspection, and predator avoidance. Such conditions could promote the development of kin‐biased interactions and kin recognition through environmental or phenotypic cues (Hepper 1986; Krause and Ruxton 2002; Tang‐Martinez 2001). Genetic analyses of juvenile bull sharks in Fijian rivers have demonstrated the presence of full and half‐sibling pairs, both within the same cohort and across successive cohorts within the same river system (Glaus 2019), indicating that related individuals co‐occur and overlap spatially during these formative stages. This supports the hypothesis that kin associations could be established early in life. However, bull sharks undergo ontogenetic habitat shifts and disperse widely as they mature, moving into coasts and channel systems where encounter rates with kin may decline. Whether kin relationships formed during early life stages translate into social bonds that persist into adulthood remains unknown. Social preferences have been documented in adult bull sharks (Bouveroux et al. 2021; Marosi et al. 2026b), yet the mechanisms structuring these preferences are only just beginning to be investigated.

Here we test whether both genetic relatedness (a continuous measure of shared ancestry) and pedigree‐based kinship (a categorical measure of inferred pedigree relationship) influence the social structure and directed social interactions of adult bull sharks, a process we refer to as “kinfluence.” The Shark Reef Marine Reserve is a provisioned site where large numbers of adult bull sharks encounter one another repeatedly, and these conditions facilitate the formation of social relationships that differ in strengths (Marosi et al. 2026b). Using DArTseq single nucleotide polymorphism (SNP) genotyping, social network analysis and 6 years of behavioral observations collected on this aggregation, we examined whether genetically related individuals associate and socially interact more strongly than expected by chance. Specifically, we tested whether higher genetic relatedness predicts proximity‐based associations and directed social interactions, and whether close kin categories including full‐ and half‐sibling dyads exhibit elevated levels of association and social interaction relative to non‐kin. If kin‐mediated processes remain important drivers of sociality, we predict a finding of “kinfluence.” This study therefore provides the first direct test of kin‐structured sociality or “kinfluence” in a wild adult population of bull sharks.

Previous analysis of this population demonstrated a non‐random, moderately connected social network without distinct community structure and identified age‐class, sex, size, and personality as important drivers of social relationships (Marosi et al. 2026b; Marosi et al. 2026c). This provided a rare opportunity to test whether kinship represents an additional driver of adult social structure and sociality.

## 2 Materials and Methods

### 2.1 Study Location

The study site is the Shark Reef Marine Reserve (SRMR) in Fiji, located off the southern coast of the main island of Viti Levu (18°17′55.86″ S 178°01′12.86″ E). SRMR is a National Marine Park, stewarded by a single eco‐tourism dive operator who has conducted provisioned shark dives for over twenty‐five years, ensuring its protection through financial compensation to the Village owning the traditional fishing rights (Brunnschweiler and Earle 2006). Sharks are provisioned at depths of either 30 m, 25 m, or 15 m, with tuna heads (*Thunnus albacares, Thunnus obesus*, and *Thunnus alalunga* ). The continued provisioning has yielded an artificial aggregation of sharks and created a uniquely accessible and reliable study system, one where not only fine‐scale social behavioral data can be collected, but genetic sampling can also be conducted on a broad scale. Since 2002, temporary aggregations of bull sharks have increased from 6 to 8 individuals per dive to averages between 35 and 45 individuals year‐round, with austral winter averages of 60–70 individuals. The bull sharks are not resident to the SRMR; they exhibit larger scale movement patterns along the southern coast of Viti Levu and beyond which can include SRMR stops irrespective of provisioning (Brunnschweiler and Barnett 2013). The presence and usage of discrete sites along the coastline vary for individual sharks depending upon the respective resources offered and thus potential fitness benefits each site can confer (Brunnschweiler and Baensch 2011; Castro 1983; Heupel et al. 2007; Simpfendorfer and Milward 1993; Speed et al. 2010). As individuals exhibit variation in site fidelity and home range, absence from the Reserve can persist for weeks, months, or even years (Brunnschweiler and Barnett 2013). Pregnant females are absent for the portions of the parturition season (November—December), and sexually active females and males will visit intermittently during the mating season (December—February). The visiting aggregation exhibits an 85% to 15% female‐biased ratio (Shark Reef Marine Reserve database) and is composed primarily of adults (sexually mature) with a small number of advanced adults (post‐reproductive), and an even smaller number of subadults (Marosi et al. 2026b). The age‐classes of the study aggregation were determined pursuant to the methods described in Marosi et al. (2026b).

### 2.2 Subject Identification

Since 2003, the scientific database of the SRMR has named and cataloged individual sharks belonging to eight different species. The database currently contains 180 named individual bull sharks. Individuals are named pursuant to a protocol where they must be easily and consistently identifiable. As a condition to entry in the database, a shark must exhibit some distinguishing feature such as scarring, deformities, missing or damaged fins, or a unique coloration pattern (Brunnschweiler and Baensch 2011). An additional 50 unnamed bull sharks were ID tagged according to the protocol outlined in Marosi et al. (2026b), and a subset of those individuals were used in the current study.

### 2.3 Social Data Collection, Network Construction and Social Interactions

**FIGURE 1:**
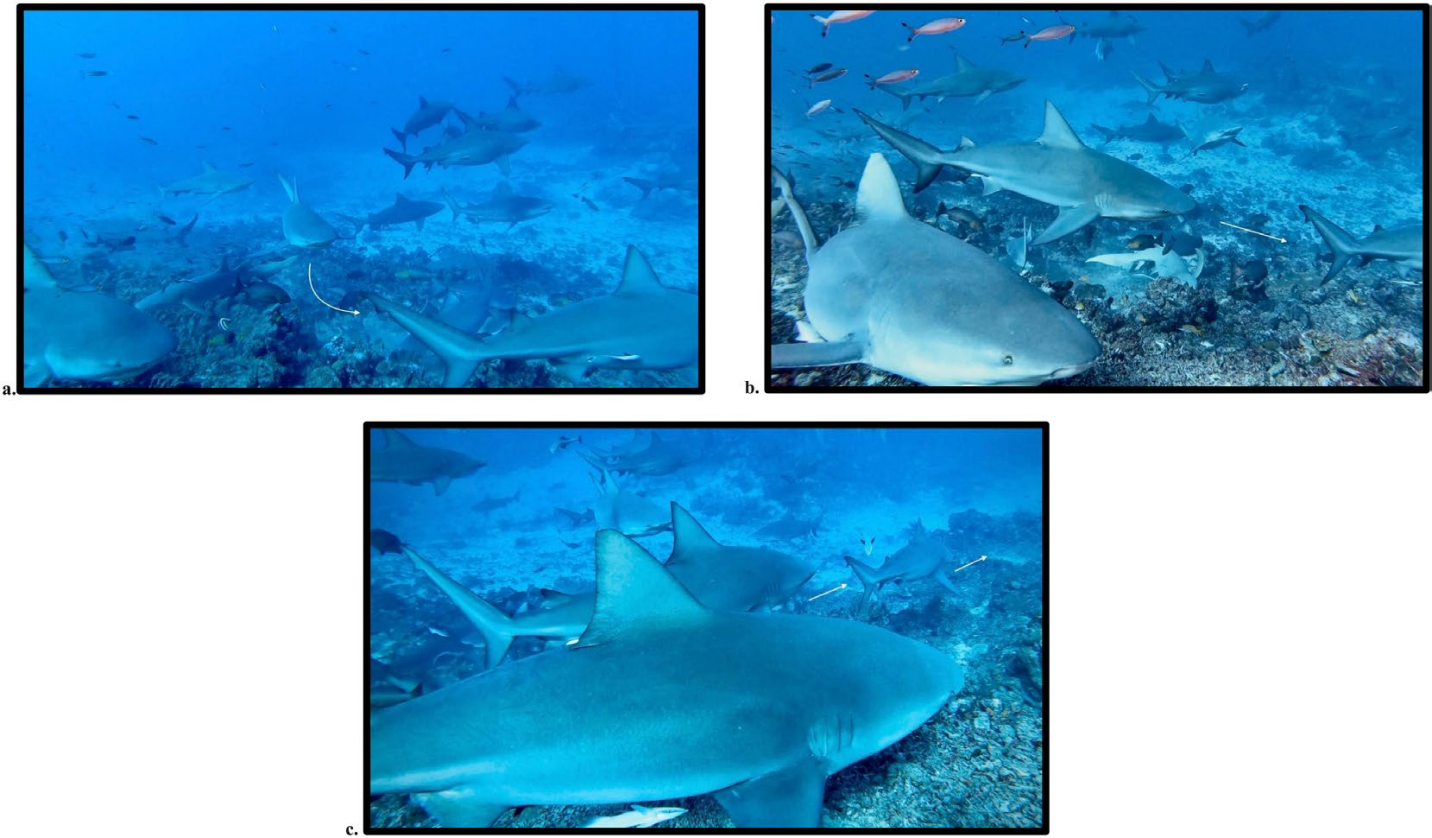
(a–c) social interactions between male dyads “Mogul” and “Chunky” identified as first cousin kin by all three optimal relatedness estimators, and identified as HS (half‐siblings) by COLONY Pedigree reconstruction. (a) Mogul exhibits a “join” behavior aligning himself behind Chunky. (b) Mogul “follows” Chunky who is in the “leader” position. (c) Mogul continues to follow Chunky, keeping a straight‐line swim configuration.

Social data used in this study were collected between 2018 and 2023 on observational dive surveys, and are described in detail in Marosiet al. (2026b). Briefly, close associations among dyads were defined using the “chain rule,” where individuals within one body length of each other and linked through nearest neighbors were recorded at one‐minute intervals (Ramos‐Fernández et al. 2009; Rimbach et al. 2015; Wolf and Weissing 2012). Association strength was calculated using the Simple Ratio Index (SRI) (Cairns and Schwager 1987). Social interactions between dyads were recorded during focal follows and included behaviors such as “join,” “lead,” “follow,” “parallel swim” and “turn back” (Myrberg Jr and Gruber 1974) (Figure 1a–c). These behaviors were coded in BORIS animal behavioral software (Friard and Gamba 2016) and used to generate a dyadic sociability matrix. Network construction and permutation procedures used to test for non‐random association and social interaction patterns are described fully in Marosi et al. (2026b).

### 2.4 Tissue Sample Collection

**FIGURE 2:**
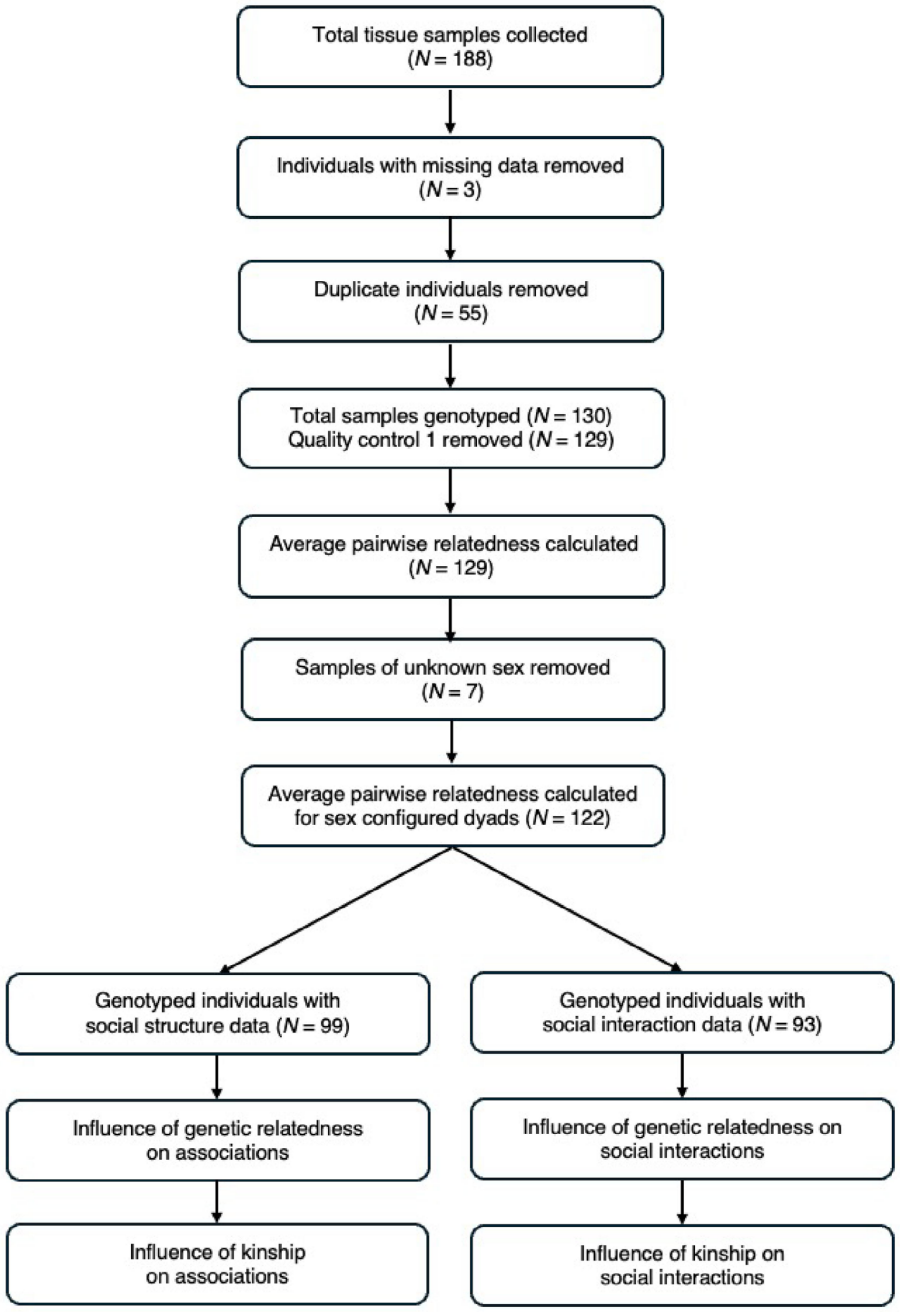
Flow chart of methods for *N* = 188 genetically sampled bull sharks, 129 genotyped individuals, *N* = 99 genotyped with social structure data and *N* = 9*3* genotyped with social interaction data.

Tissue samples were collected underwater during the provisioned tourism dives, using a small pneumatic speargun outfitted with a custom threaded shaft. The base and biopsy probe were crafted for the speargun; the probe contained a rubber stopper to prevent it from penetrating too deeply into the subject. Tissue samples (dermis and muscle) were collected using biopsy probes, preserved in 99% ethanol, and stored at −20C until analysis. Sample metadata (date and individual identity, where known) were recorded at the time of collection. A total of 188 samples were available for analysis, comprising 89 samples collected by NDM between 2021 and 2023 and 99 archival samples collected at the same site between 2012 and 2020 by Beqa Adventure Divers staff, representing a mixture of identified and unidentified individuals (Figure 2). The unknown individuals were included to increase sample size and improve the accuracy of estimating allele frequencies for the population.

### 2.5 Data Analyses

#### 2.5.1 Single Nucleotide Polymorphism Discovery and Genotyping

A total of 188 tissue samples each consisting of a slice of dermis between 10 and 15 mg in wet weight were sent to Diversity Arrays Technology (DArT Pty Ltd., Canberra, Australia) for SNP discovery and genotyping using the DArtseq platform. Genomic DNA was extracted by DArT using the NucleoMag Tissue kit (Macherey‐Nagel). Tissue was lysed in 50 μL T1 buffer supplemented with 6.25 μL proteinase K, and digested overnight at 60°C. Lysates were clarified by centrifugation (10 min 3000 rpm) and DNA was captured on NucleoMag B‐beads (6 μL beads in 90 μL MB2) under continuous agitation. Wash steps and DNA elution were automated on Tecan T100 liquid handling platform using a 96‐tip head and a DArT proprietary script.

Library preparation, complexity reduction and SNP genotyping were performed following the standard DArTseq protocol (Georges et al. 2018; Kilian et al. 2012). Genomic DNA was digested using the restriction enzymes Pstl and Sphl, followed by ligation of adaptors with compatible restriction enzyme overhangs (Sansaloni et al. 2011). The Pstl‐compatible forward adaptor incorporated the Illumina flowcell attachment sequence, sequencing primer sequence and a staggered barcode region to enable multiplexing (Elshire et al. 2011) while the Sphl‐compatible reverse adaptor contained the corresponding flowcell attachment region and Sphl‐compatible overhang.

Multiplexed libraries were sequenced on an Illumina NovaSeq 6000 platform. Sequencing yielded an average of 2,020,863 reads per sample (SD = 620,790; CV = 30.72%). Following demultiplexing and quality filtering, an average of 432,709 unique sequences per sample was retained and used for downstream SNP calling. Raw sequence data were processed using DArT proprietary analytical pipelines for marker calling and quality filtering.

#### 2.5.2 SNP Filtering

Following DArT's initial SNP calling and quality control pipeline, 39,986 polymorphic SNPs were obtained. SNP filtering was performed using the function *fl.filter.reproducibility* from the package “dartR.base” (Mijangos et al. 2022), applying the DArTseq locus‐level reproducibility metric (RepAvg), calculated from technical replicates genotyped independently from the same DNA extracts as the proportion of concordant genotype calls across replicate runs. SNPs were retained if they met the following criteria: (i) polymorphic loci, (ii) call rate ≥ 0.95, (iii) reproducibility (RepAvg) ≥ 0.99, (iv) mean read depth ≥ 10, (v) minor allele frequency (MAF) ≥ 0.008 (equivalent to a minor allele count (MAC) of ≥ 3), and (vi) no significant deviation from Hardy–Weinberg equilibrium (*p* ≥ 0.05). To minimize bias arising from genotype uncertainty, a relatively strict minimum read depth threshold of 10× was applied. Although this filter reduces the number of retained loci, higher read depth substantially decreases the probability of stochastic allelic dropout and low‐confidence genotype calls. This is particularly important for relatedness and pedigree analysis, where even low levels of genotyping error can bias relatedness estimates and attenuate correlations among dyads. To reduce marker redundancy one SNP was removed from pairs showing high linkage disequilibrium (*r* ^2^ > 0.2), low Hamming distance between sequence tags (< 0.2), or representing secondary SNPS within the same sequence fragment (Table 1). Individuals with more than 50% of missing data (UM_5, UF_17, UF_13) were also excluded. After filtering, the dataset comprised 3919 SNPs genotyped in 185 bull shark individuals (see Table 1 and Figure 2).

**TABLE 1:** Filters and their thresholds used on the SNP dataset.

Filter	Threshold	Loci filtered	Loci remaining
Monomorphic loci	NA	14,101	25,885
Missing data by site (callrate)	< 0.95	5623	20,262
Reproducibility (RepAvg)	< 0.99	8376	11,886
Read depth	< 10	4875	7011
Minor allele frequency (MAF)	< 0.008	1389	5622
Secondaries	NA	362	5260
Hardy–Weinberg Equilibrium	< 0.05	253	5007
Linkage disequilibrium	> 0.2	479	4528

#### 2.5.3 Identification of Duplicated Individuals

Duplicate samples originating from the same individual were identified following genotyping and removed prior to all relatedness and kinship analyses to avoid inflation of identity‐by‐descent estimates. To identify duplicate individuals in the dataset, the function *gl.report.replicates* from the CRAN package dartR.base was used (Mijangos et al. 2022). A total of 55 duplicated individuals were identified (Figure 2).

#### 2.5.4 Heterozygosity Filtering

To assess whether individual level heterozygosity or inbreeding could bias relatedness estimates and to screen for potential cross sample contamination, individual level quality control was implemented following Keller et al. (2011). Allele‐frequency‐weighted inbreeding coefficients (F_alt) were calculated in GCTA v1.94 (Yang et al. 2011) using the ‐‐ibc option which weights homozygous loci by the inverse of allele frequency. Excess homozygosity relative to Hardy–Weinberg expectations (F_h) was calculated for each individual using PLINK v1.9 (Purcell et al. 2007) with the ‐‐het option. Observed heterozygosity per individual was additionally estimated using the function gl.report.heterozygosity implemented in the R package *“dartR.base”* (Mijangos et al. 2022). Distributions of individual heterozygosity and inbreeding coefficients were examined to identify individuals with excessively negative inbreeding values, which can be indicative of sample contamination or genotyping artifacts. One individual (“Oceania”) showed extreme heterozygote excess and strongly negative inbreeding estimates consistent with potential sample contamination and was therefore removed as a conservative filtering step (Figure 2).

#### 2.5.5 Genetic Diversity and Inbreeding Coefficient

To assess genetic variation, observed heterozygosity (Ho), unbiased expected heterozygosity under random mating (uHe; corrected for sample size), and the inbreeding coefficient (FIS) were calculated using the *gl.report.heterozygosity* function in the R package “dartR” (Mijangos et al. 2022). Confidence intervals (CIs) were estimated using 2000 bootstrap replicates and calculated following the bias‐corrected and accelerated (BCa) method (DiCiccio and Efron 1996).

#### 2.5.6 Relatedness Estimation and Simulations

It has been recommended that when estimating relatedness coefficients, simulations are conducted to select an optimum measure that considers the specific characteristics of the focal population (e.g., Foroughirad et al. 2019). Using the R package “related” (Pew et al. 2015), the dataset's allele frequencies were, therefore, used to simulate pairs of individuals of known relatedness to test a broad range of twelve relatedness estimators. Relatedness coefficients were then calculated for simulated pairs of individuals at a range of relatedness (i.e., full‐sibling, parent‐offspring = 0.5; half‐sibling = 0.25; first cousin, aunt/uncle‐niece/nephew = 0.125; second cousin = 0.03125; unrelated = 0, with 100 replicates of each). The Root Mean Squared Error (RMSE) between observed and expected values for pairs of simulated individuals of known relatedness was used to measure the accuracy of relatedness estimators. The RMSE quantified the average deviation between the estimated relatedness values and the true (expected) relatedness values across all pairs. A lower RSME indicates a more accurate estimator, as it implies that the estimated values are closer to the true values on average. The optimum estimators are those having the highest correlation with the expected values, the lowest variance and lowest RSME. Once the optimum estimators were determined, they were calculated for the 129 genotyped sharks using the R package “related” (Pew et al. 2015). To investigate how pairwise genetic relatedness between same sex individuals differs, or could be biased, three binary matrices (0,1) were constructed that consisted of same sex dyads (FF, MM) and opposite sex dyads (FM) after removal of seven individuals for which sex was unknown (UKN) (*n* = 122) (Figure 2).

Mantel tests in the “vegan” package (Oksanen et al. 2025) of R for 20,000 permutations to test for correlations with the relatedness matrix. Average pairwise relatedness was also calculated for each sex‐configured dyad across the three optimum estimators. To evaluate whether sex‐specific configurations of average pairwise relatedness were due to chance, 10,000 permutations were run, and sex labels were randomly shuffled across individuals with dyads re‐classified as FF, FM, or MM. Observed mean relatedness and the differences between the sex‐configured dyads were compared to the null distributions to generate two‐sided *p*‐values. Significant deviations indicate that the observed patterns are unlikely to result solely from random assignment.

#### 2.5.7 Kinship Reconstruction Using COLONY

Parentage and sibship inference were conducted in COLONY v2.0 (Jones and Wang 2010a, 2010b) using the gl.run.colony wrapper implemented in the “dartR.captive” package (Gruber et al. 2018; Mijangos et al. 2022). COLONY was run under a polygamous mating system for both sexes, assuming no inbreeding, with a medium run length, the Full‐Likelihood model, and medium full‐likelihood precision.

Genotypes were analyzed under three alternative SNP‐filtering strategies to evaluate the robustness of pedigree reconstruction to marker filtering. All datasets were first subjected to baseline filtering, including removal of individuals with call rate < 0.5, removal of monomorphic loci, and removal of secondary loci. Additional locus‐level filters were then applied as follows: Stringent dataset (3827 SNPs): Hardy–Weinberg equilibrium (HWE) *p* < 0.05; linkage disequilibrium (LD) *r* ^2^ > 0.2; Hamming distance > 3 mismatches; locus call rate > 0.95; reproducibility > 0.99; read depth > 10; minor allele count (MAC) > 3. Relaxed dataset (7017 SNPs): HWE *p* < 0.05; LD *r* ^2^ > 0.2; Hamming distance > 3 mismatches; locus call rate > 0.90; reproducibility > 0.98; read depth > 5; MAC > 2. Relaxed dataset without HWE/LD/Hamming filtering (9410 SNPs): locus call rate > 0.90; reproducibility > 0.98; read depth > 5; MAC > 2.

Across filtering strategies, COLONY outputs were compared on a single spreadsheet to identify consistent full‐sibling and half‐sibling assignments retained for downstream kinship analysis. Importantly, no candidate parents were specified in the COLONY input. This decision reflects the biological characteristics of the study population: sampled individuals were predominantly adults, drawn from overlapping generations sampled across an extended period with unknown parental cohorts and evidence of multiple paternity. This made it very difficult/impossible to clearly define a parental generation across the entire dataset/population.

#### 2.5.8 Genetic Relatedness, Social Structure and Social Interactions

Unless otherwise stated, all analysis was computed in R.Studio v. 4.4.3 (R Core Team 2025) and social data was visualized using “igraph” within R. Studio (Csárdi et al. 2024; Csárdi and Nepusz 2006). To investigate the role of genetic relatedness in shaping social structure and social interactions within the visiting population of bull sharks, analyses were restricted to individuals for which both genetic and behavioral data (social data collected in Marosi et al. 2026b) were available (Figure 2). For association analyses, the dataset was restricted to individuals with both genetic and association data (*n* = 99), while analyses of social interactions were restricted to individuals with both genetic and interaction data (*n* = 93). Individuals were excluded from analyses if they were not present at the study site during dedicated sampling periods, could not be biopsied due to limited proximity to the observation wall, or lacked corresponding social behavioral data.

The relationship between genetic relatedness and social structure was evaluated using Mantel two‐tailed tests (Mantel 1967) with 9999 permutations implemented using the Pearson correlation coefficient in the community ecology package “vegan” (Oksanen et al. 2025). Tests were conducted to assess the correlation between relatedness and association matrix (SRI; Simple Ratio Index) to see whether more closely related individuals interacted more frequently. The same analytical framework was applied to the dyadic sociability matrix, which quantified the weighted number of observed social interactions between dyads.

Mantel tests were first performed on the entire sample of bull sharks and then separately for female and male subjects to examine potential sex‐specific patterns. For association analyses the subset included females (*n* = 81) and males (*n* = 18), while social interaction analyses included females (*n* = 74) and males (*n* = 19). Because opposite sex (female–male) dyads form a rectangular matrix (females × males), a standard Mantel test cannot be applied directly as it requires square symmetric matrices. Female–male matrices were therefore extracted from the relatedness and SRI matrices and vectorized, and the Pearson correlation between matrices was calculated. Significance was assessed using 9999 permutations where female and male labels in the relatedness matrix were independently permuted while the SRI matrix was held constant, generating a null distribution of correlations. This Mantel‐style permutation procedure was implemented using base R functions and the “dplyr” package (Wickham et al. 2026) for data preparation.

#### 2.5.9 Pedigree‐Based Kinship, Social Structure and Social Interactions

To complement analyses based on continuous pairwise relatedness, we conducted an additional analysis using discrete kinship categories inferred through pedigree reconstruction. Sibship assignments were taken from the *relaxed* COLONY run, which allows for realistic genotyping error while avoiding the overly conservative assumptions of the stringent run and the increased false‐positive risk associated with the relaxed‐noHW configuration. Dyads were retained as full‐ or half‐siblings only when the posterior probability of sibship was ≥ 0.80, following Wang (2017).

Two dyadic kinship matrices were constructed to test the influence of close pedigree kinship on social structure and social interactions. In the first, a binary kinship matrix was generated in which full and half‐sibling dyads were treated equally (FS = 1, HS = 1), with all other dyads coded as 0. In the second, a weighted kinship matrix was constructed to reflect differences in expected relatedness between pedigree categories (FS = 1, HS = 0.5; non‐kin = 0). For each type, an *N* × *N* symmetric matrix was generated matching the individual order of the social networks.

Relationships between pedigree‐based kinship, association strength and social interaction strength were evaluated using Mantel tests implemented in the R package “vegan,” using Pearson correlations with 9999 permutations. These analyses were conducted separately for the association network (SRI matrix) and the dyadic sociability matrix, which quantified the weighted number of observed social interactions between dyads. Networks were restricted to individuals for which both genetic and behavioral data were available (associations: *n* = 99; social interactions: *n* = 93) (see Figure 2).

## 3 Results

### 3.1 SNP Genotyping, Genetic Diversity and Inbreeding Coefficient

DArTseq SNP genotyping was successfully used to generate a panel of ~4000 high‐quality independent SNPs across 188 tissue samples that distinguished 129 bull sharks visiting the Shark Reef Marine Reserve in Fiji. The observed heterozygosity (*H*_o_ = 0.216) closely matched the unbiased expected heterozygosity (u*H*_e_ = 0.216), indicating a moderate level of genome‐wide genetic diversity and values consistent with Hardy–Weinberg expectations. The inbreeding coefficient (*F*_IS_ = −0.001 ± 0.0012 SE) was effectively zero. These results indicate the absence of detectable inbreeding and a negligible excess of heterozygotes.

### 3.2 Relatedness Estimators and Simulations

Across all the simulations the mean RSME, varied between 0.025592 and 0.015879, the mean variance also varied between 0.000240 and 0.000694. There were considerable differences in how the coefficients behaved (Tables S1 and S2). Three maximum likelihood measures (Milligan 2003; Wang 2007; Wang 2022) consistently demonstrated the lowest combinations of RSME and variance. These were considered optimal for this dataset and all three were utilized in subsequent analyses (Table S3).

### 3.3 Average Pairwise Relatedness

**FIGURE 3:**
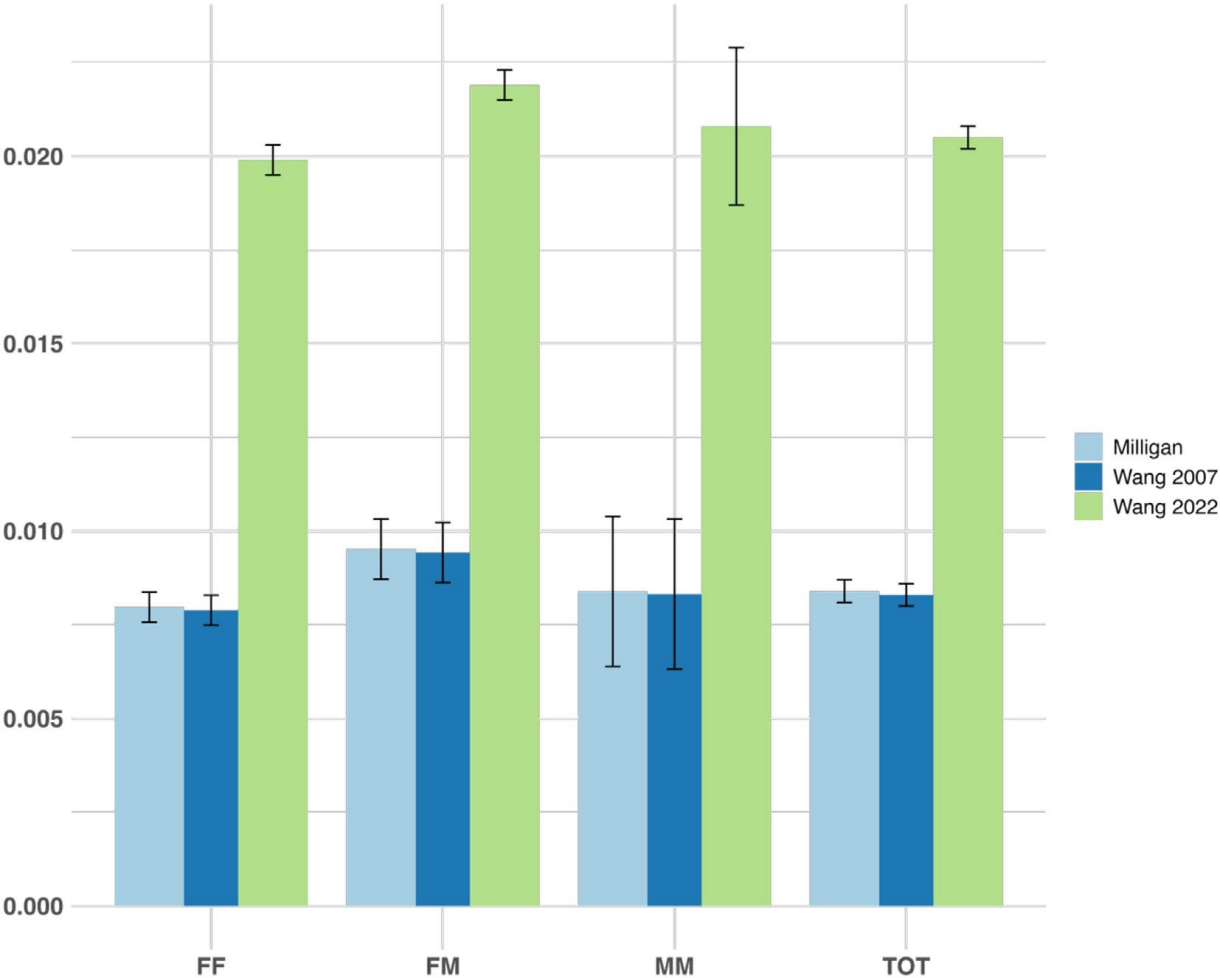
Average pairwise genetic relatedness in 122 bull shark individuals (±SE) for female–female (FF), male–male (MM), and female–male (FM) dyads, and in 129 bull shark individuals (TOT) based on three optimum relatedness estimators: Milligan, Wang (2007), and Wang (2022). Bars represent average relatedness values for each dyad type calculated using each estimator with error bars signifying standard error. (FF dyads) *n* = 5150; (MM dyads) *n* = 210; (FM dyads) *n* = 2121; (TOT dyads) *n* = 8256.

Within the study population of genotyped bull sharks (*n* = 129) visiting the Shark Reef Marine Reserve, analyses using the three optimal relatedness estimators revealed relatively low levels of average pairwise relatedness across all dyads (*n* = 8256): Wang (2007) *r* = 0.0083 ± 0.0003, Milligan *r* = 0.0084 ± 0.0003 and Wang (2022) *r* = 0.0205 ± 0.0003 (Figure 3). Across all estimators, average pairwise relatedness was higher in mixed sex (FM) dyads followed by male–male (MM) and female–female (FF) dyads (Figure 3; Table 2). Permutation tests comparing observed average relatedness to a null distribution generated by random sex assignment showed that FM and FF dyads differed significantly from null expectations, whereas MM dyads did not. The deviation from the null mean was small (△*r* ≈0.0015–0.002) but statistically significant for FM and FF dyads (Wang 2007: *p* = 0.0440 and 0.0388; Milligan, *p* = 0.0422 and 0.0370; Wang 2022: *p* = 0.0202 and 0.0214 (Table 2)).

**TABLE 2:** Observed average pairwise relatedness (*r* ± SE) among sex‐configured dyads based on individuals of known sex (*n* = 122; 7381 dyads).

Dyad type by relatedness estimator	Average pairwise relatedness	SE	Perm *p*
Wang 2007 FF	0.00784	±0.0003	**0.0388**
Wang 2007 FM	0.00933	±0.0007	**0.0440**
Wang 2007 MM	0.00832	±0.0020	0.9834
Milligan FF	0.00792	±0.0003	**0.0370**
Milligan FM	0.00942	±0.0007	**0.0422**
Milligan MM	0.00838	±0.0020	0.9790
Wang 2022 FF	0.01991	±0.0003	**0.0214**
Wang 2022 FM	0.02186	±0.0007	**0.0202**
Wang 2022 MM	0.02084	±0.0020	0.8550

*Note:* Significant values indicate dyad types with average relatedness greater than expected under random sex assortment. FF dyads (*n* = 5050); FM dyads (*n* = 2121); MM dyads (*n* = 210). Bold values indicates which *p* values are significant.

### 3.4 Pedigree Reconstruction and Kin Composition

**FIGURE 4:**
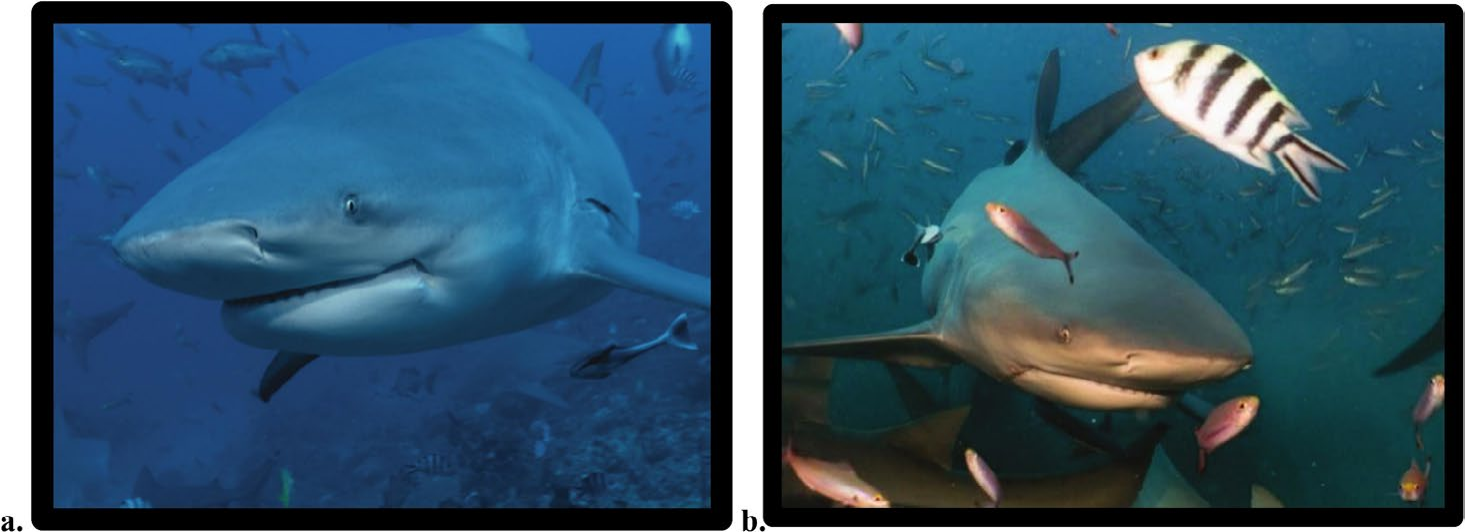
(a, b) Close kin female bull shark dyad identified as PO/FS (parent‐offspring/full siblings) by all three relatedness estimators and as FS (full siblings) by COLONY pedigree reconstruction. (a) female bull shark “Crook,” (b) female bull shark “Vendetta.” Image (a) provided by Mike Neumann and image (b) provided by Natasha D. Marosi.

Pedigree reconstruction using COLONY identified close‐kin dyads within the genotyped sample under the relaxed filtering strategy (posterior probability > 0.80). Across all genotyped individuals, COLONY inferred 11 full‐sibling dyads and 46 half‐sibling dyads (Figure 4a,b; Table S4).

### 3.5 Genetic Relatedness, Social Structure and Social Interactions

#### 3.5.1 Genetic Relatedness and Social Structure

**FIGURE 5:**
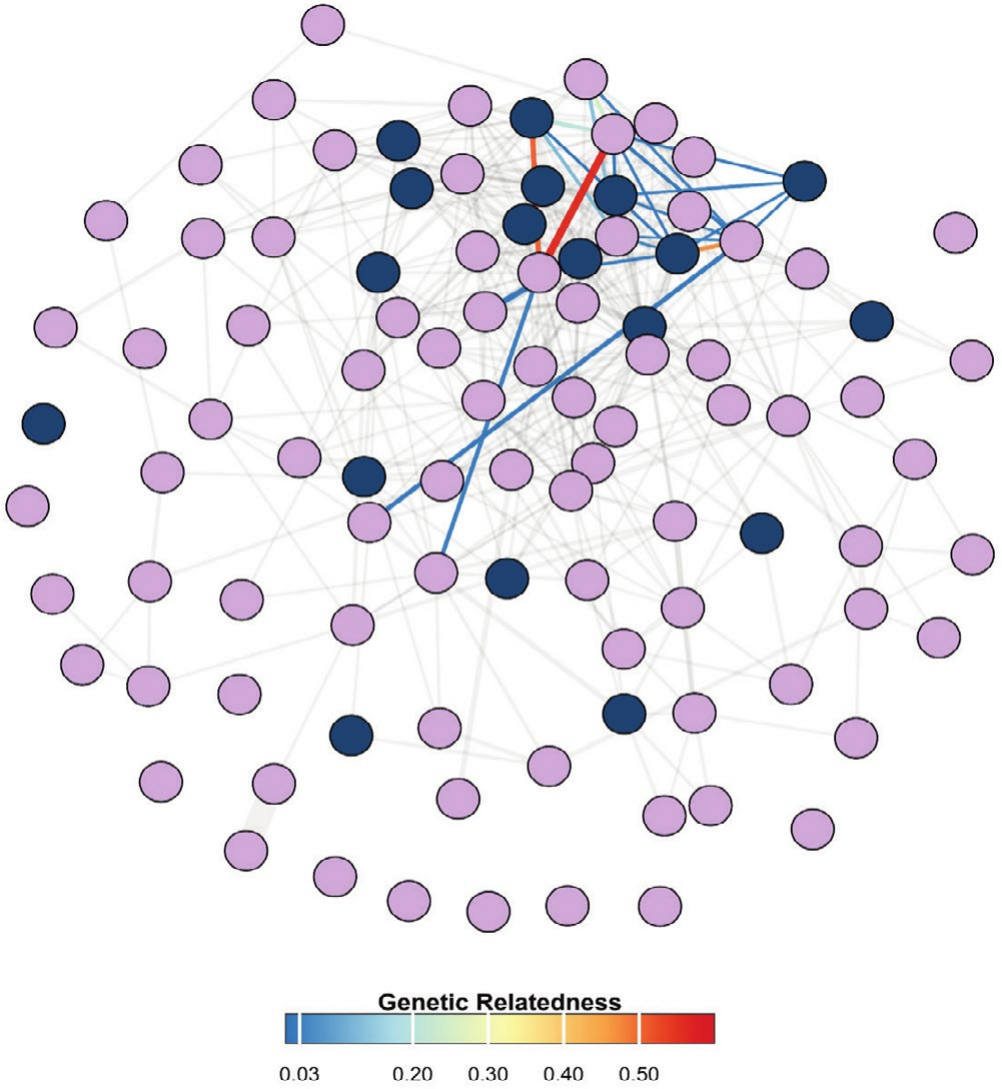
Bull shark association network (*n* = 99) with genetic relatedness overlaid as a continuous gradient. The network is based on the top 30% of pairwise Simple Ratio Index values (SRI) to emphasize stronger association patterns. Purple nodes represent female bull sharks; darker blue nodes represent male bull sharks. Edge width is scaled to SRI values. Edge color overlay represents pairwise genetic relatedness (*r*) mapped onto a continuous gradient from fourth order relations (blue, *r* ≥ 0.03125) to first order relations (red, *r* ≥ 0.50). Edges below the relatedness threshold (non‐kin) are shown in gray.

The subset of 99 genotyped individuals with corresponding association data were analyzed to examine the influence of genetic relatedness on associations (Figure 5). Mantel tests conducted using the three optimal relatedness estimators and SRI matrices (*n* = 99; 4851 possible dyads) revealed weak positive correlations between relatedness and association strength across all estimators, although only one estimator produced a statistically significant result at the 0.05 level (Table 3). Analysis of female dyads (FF) showed a similar pattern of weak positive correlations across estimators, whereas male (MM) and mixed‐sex dyads (FM) exhibited little to no correlation between genetic relatedness and association strength (Table 3).

**TABLE 3:** Correlation coefficients and significance of genetic relatedness on associations for total sample and for female and male subset yielded by two‐tailed Mantel test.

Estimator	Total *r*	Total *p*	FF *r*	FF *p*	MM *r*	MM *p*	FM *r*	FM *p*
Wang2007	0.021	0.057	0.021	0.075	0.031	0.332	0.015	0.568
Milligan	0.021	0.058	0.021	0.085	0.030	0.302	0.015	0.572
Wang2022	0.027	**0.025**	0.028	**0.040**	0.004	0.491	0.021	0.408

*Note:* Total subset *n* = 99; females *n* = 81; males *n* = 18. Bold values indicates which *p* values are significant.

#### 3.5.2 Genetic Relatedness and Social Interactions

**FIGURE 6:**
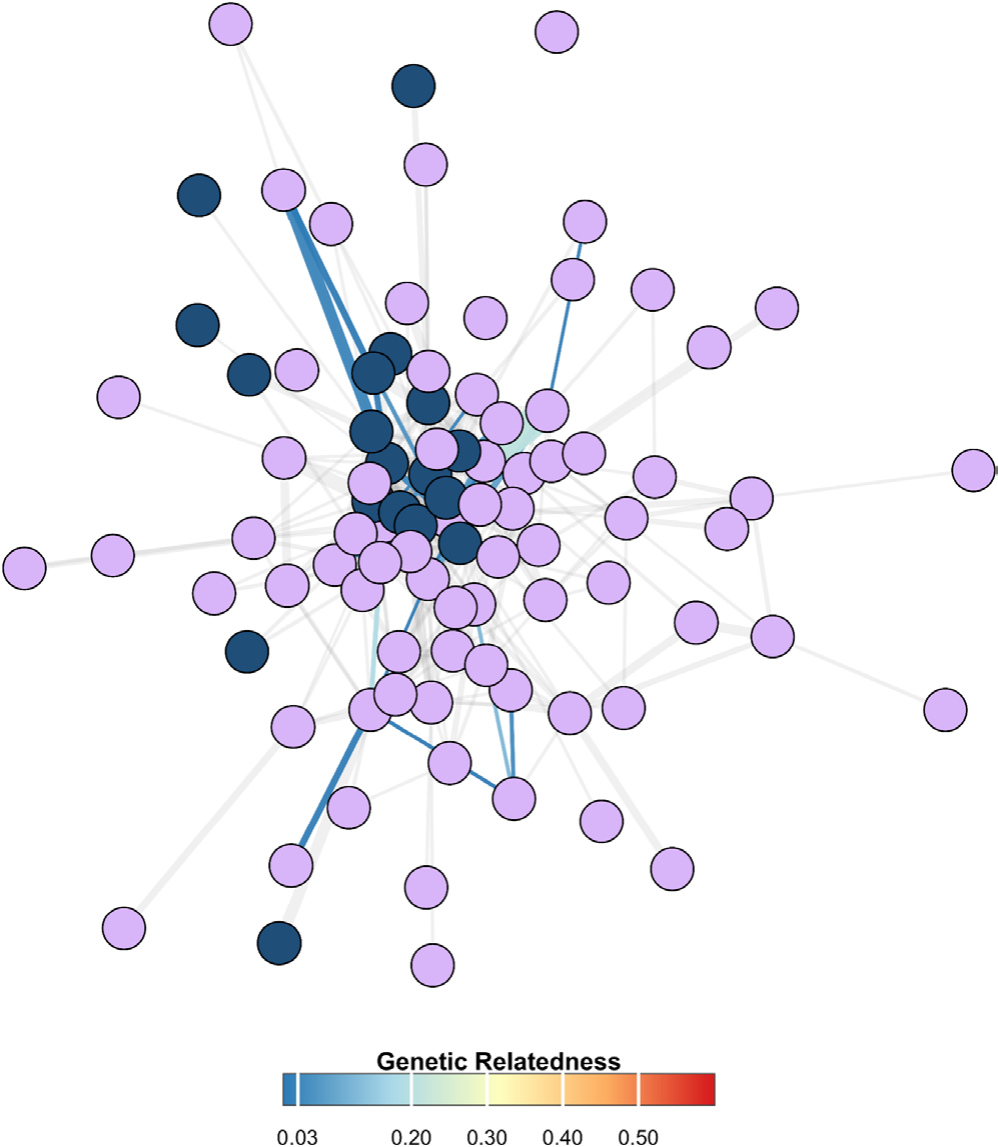
Bull shark social interaction network (*n* = 93) with genetic relatedness overlaid as a continuous gradient. Lighter blue nodes represent female bull sharks; darker blue nodes represent male bull sharks. Edge width reflects total strength of social interactions between dyads, calculated from a weighted combination of observed social behaviors. Edge color overlay represents pairwise genetic relatedness (*r*) mapped onto a continuous gradient from fourth order relations (blue, *r* ≥ 0.03125) to first order relations (red, *r* ≥ 0.50). Edges below the relatedness threshold (non‐kin) are shown in gray.

Mantel tests run on the three optimal relatedness coefficients, and the dyadic sociability matrix (*n* = 93; 4278 possible dyads) generally reveal a slight positive correlation which is statistically significant (Figure 6; Tables S3, S4). Analysis of female dyads (FF) slightly weakened the strength of the positive correlation and none of the results were statistically significant (Table 4). Analysis of male dyads (MM) revealed a significant, stronger positive correlation across all three estimators (Table 4). Analysis of mixed‐sex dyads (FM) revealed no correlation between genetic relatedness and association strength.

**TABLE 4:** Correlation coefficients and significance of genetic relatedness on social interactions for total sample and for female and male subset yielded by two‐tailed Mantel test.

Est	Total *r*	Total *p*	FF *r*	FF *p*	MM *r*	MM *p*	FM *r*	FM *p*
Wang2007	0.03318	**0.0339**	0.008774	0.2188	0.2434	**0.0119**	−0.009	0.709
Milligan	0.03322	**0.0345**	0.009127	0.2146	0.2431	**0.0119**	−0.010	0.700
Wang2022	0.02933	**0.0439**	0.008208	0.231	0.2197	**0.0159**	−0.013	0.591

*Note:* Total subset *n* = 93; females *n* = 74; males *n* = 19. Bold values indicates which *p* values are significant.

#### 3.5.3 Categorical Kinship and Social Structure

**FIGURE 7:**
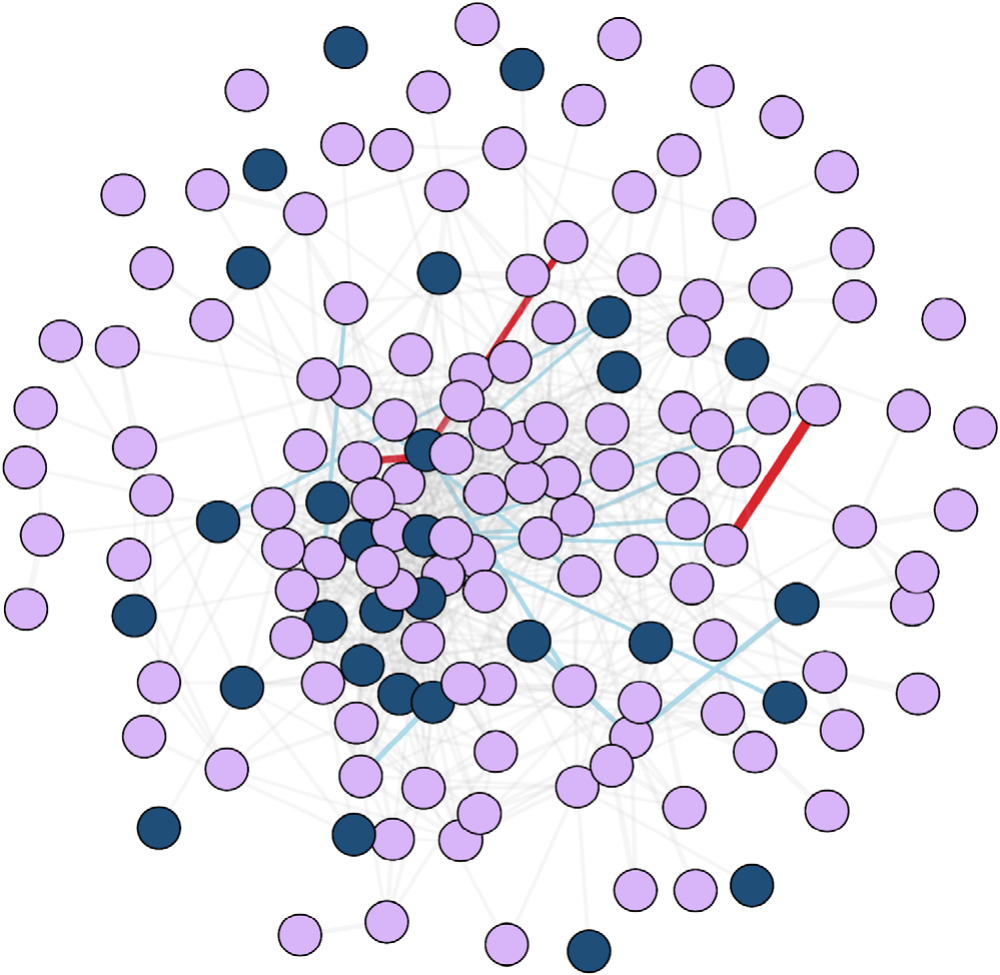
Bull shark association network with COLONY inferred kinship overlay, based on top 30% of pairwise SRI values to emphasize stronger network connections. Edge widths are scaled to SRI values. Lilac nodes represent female bull sharks, navy nodes represent male bull sharks, red edge overlay represents full sibling dyads, light blue edge overlay represents half sibling dyads, gray edges represent non‐kin dyads.

Out of the subset of 99 genotyped individuals with association data (4851 possible dyads), full‐sibling dyads (FS) were extremely rare (*n* = 6), while half‐sibling dyads were also uncommon (*n* = 31 dyads). When FS and HS dyads were treated equally in a binary kinship matrix, no relationship between kinship and association strength was detected. However, when kinship was weighted to reflect expected relatedness differences between pedigree categories (FS = 1, HS = 0.5), a positive significant relationship was found (Mantel *r* = 0.023, *p* = 0.0499) (Figure 7, Table 5).

**TABLE 5:** Mantel correlations between pedigree‐based kinship and association and social interaction networks using binary (FS = 1, HS = 1) or weighted (FS = 1, HS = 0.5) kinship matrices.

Network	Kin coding	Mantel *r*	*p*
Association	Binary (FS = 1, HS = 1)	0.015	0.106
Association	Weighted (FS = 1, HS = 0.5)	**0.023**	**0.049**
Social Interaction	Binary (FS = 1, HS = 1)	0.006	0.297
Social Interaction	Weighted (FS = 1, HS = 0.5)	0.002	0.376

*Note:* Association subset *n* = 99; social interaction subset *n =* 93. Bold values indicates which *p* values are significant.

#### 3.5.4 Categorical Kinship and Social Interactions

**FIGURE 8:**
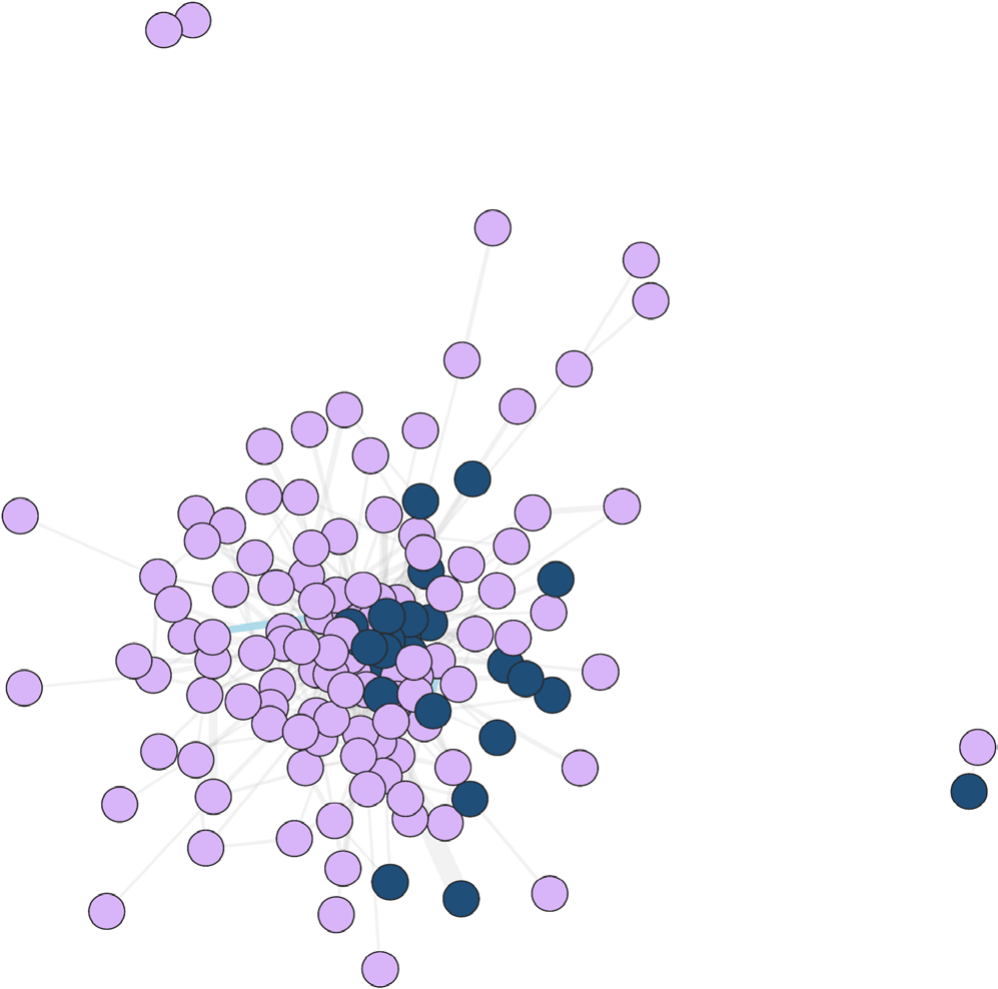
Bull shark social interaction network with COLONY inferred kinship overlay. Edge width reflects total strength of social interactions between dyads, calculated from a weighted combination of observed social behaviors. Lilac nodes represent female bull sharks, navy nodes represent male bull sharks, red edge overlay represent full sibling dyads, light blue edge overlay represents half sibling dyads, gray edges represent non‐kin dyads.

Out of the subset of 93 genotyped individuals with social interaction data (4278 possible dyads), FS dyads were extremely rare (*n* = 6), while HS dyads were also uncommon (*n* = 28 dyads). In contrast to the pattern uncovered for associations, Mantel tests revealed no relationship detected between pedigree kinship and social interaction strength for either binary or weighted kinship matrices (Figure 8; Tables 5, 6).

**TABLE 6:** Frequencies of dyadic social interactions by kinship category and dyad sex among genotyped individuals (*n* = 93; 4278 possible dyads).

Kinship category	Sex dyad	*N* dyads	Mean interactions	SD interactions	Max interactions	Total interactions
Full Sibling	FF	4	0	0	0	0
Full Sibling	MM	**0**	0	0	0	0
Full Sibling	FM	2	0	0	0	0
Half Sibling	FF	12	0.75	1.86	6	9
Half Sibling	MM	2	0	0	0	0
Half Sibling	FM	14	0.357	0.929	3	5
Non‐kin	FF	2685	0.231	1.24	20	**619**
Non‐kin	MM	169	1.88	4.15	32	**317**
Non‐kin	FM	1390	0.288	1.09	11	**401**

*Note:* Dyads were classified as full siblings, half‐siblings, or non‐kin based on COLONY pedigree reconstruction (posterior probability ≥ 0.80). For each kinship × sex category (FF, MM, FM), the number of dyads, mean, standard deviation (SD), median, and maximum interaction strength is reported as well as total number of social interactions. Bold values indicates which *p* values are significant.

## 4 Discussion

### 4.1 Overview of “Kinfluence” in the Shark Reef Marine Reserve Aggregation

Our investigation of “kinfluence” (the influence of kin on social structure) using two complementary methodological approaches on two datasets yielded mixed but biologically meaningful results. Overall, we found that while the adult bull shark aggregation visiting the Shark Reef Marine Reserve is not kin structured at the population level, genetic relatedness and categorical kinship nevertheless may contribute to aspects of both association and social interaction dynamics. Genetic relatedness exhibited weak positive relationships with association patterns in the total population and among female–female dyads across all three optimal estimators, although only one estimator reached statistical significance. The strongest relationship between genetic relatedness and social behavior was observed within fine‐scale social interaction dynamics, particularly among male–male dyads. By contrast, pedigree reconstruction and categorical kinship assignment identified very few close kin dyads in the aggregation, and the rarity of these full and half‐sibling dyads constrained the extent to which close familial relations could explain social structure at the community or dyad levels.

These findings are notable because they suggest kin‐selection may operate subtly within a wild, free‐ranging population where multiple demographic, ecological and life‐history factors play a role in shaping social behavior. Although the observed Mantel correlation coefficients were small, the detection of any kin‐based signal is itself important given that our genetic dataset represents approximately 25% of the sharks known to visit the study site (SRMR database), relatedness estimators indicated low average pairwise relatedness, and pedigree reconstruction indicated small numbers of dyads with close familial relationships. Our study therefore remains one of a very small number of investigations examining kin‐mediated sociality in elasmobranchs (see Andrews et al. 2009; Guttridge et al. 2011; Newby et al. 2014; Mourier and Planes 2021), and the first to do so in bull sharks. Importantly, our results add to a growing body of evidence that elasmobranch social systems are more complex than previously thought, even in species traditionally viewed as solitary.

### 4.2 Population Genetic Context

Genetic analysis of the Shark Reef Marine Reserve aggregation suggested moderate levels of heterozygosity. These findings are broadly consistent with those reported by Glaus et al. (2020), which analyzed an independent dataset from this system collected in earlier years. In contrast, Devloo‐Delva et al. (2023), working from a much smaller subset of the same aggregation, reported markedly lower levels of genetic diversity and greater evidence of inbreeding. Discrepancies among studies likely reflect differences in sample size, marker ascertainment and genotyping approaches, and filtering schemes, all of which are known to strongly influence heterozygosity estimates derived from reduced representation SNP datasets (Hauser et al. 2022; Jones and Wang 2010a, 2010b).

Average pairwise relatedness across the aggregation was uniformly low, indicating that this adult population is not kin‐structured at the population level. However, when dyads were partitioned by sex configuration, female–female and female–male dyads exhibited slightly higher relatedness than expected under random assortment, whereas male–male dyads did not deviate from null expectations. Although the magnitude of these differences was small, the pattern was consistent across all three relatedness estimators and suggests a weak sex‐based lineage structure within an otherwise genetically mixed aggregation.

Such a pattern is compatible with studies documenting female regional philopatry and male‐biased dispersal in viviparous sharks (Chapman et al. 2015; Laurrabaquio‐A et al. 2019; Portnoy and Heist 2012; Tillett et al. 2011). Within this study system, evidence of parturition philopatry indicates females repeatedly use the same river nurseries (Brunnschweiler et al. 2024). If female offspring exhibit natal philopatry as has been shown in the Atlantic lemon shark (Feldheim et al. 2014), maternal female full and half‐siblings would then recruit from the same nursery systems into the adult aggregation, generating the weak but significant elevation in relatedness observed among female–female dyads. The weak but significant increase in relatedness in mixed sex dyads (female–male) may be a downstream consequence of female philopatry operating in a genetically closed population. The study aggregation is part of an insular population spanning the Fijian archipelago (Glaus et al. 2020). Therefore, if related females cluster non‐randomly, it increases the likelihood that males present in the aggregation are related to those females through both maternal and paternal lines. Indeed, full and half‐sibling pairs have been identified in the same river cohorts with half‐sibling pairs in cross‐river cohorts likely sired by the same pool of male sires (Glaus 2019). These processes would elevate the probability of detecting a small but statistically significant elevation in relatedness among female–male dyads without implying strong kin structuring. Such weak lineage structure is perhaps unsurprising in this population where dispersal, ontogenetic habitat shifts and high rates of juvenile mortality are expected to progressively dilute kin associations established in early life stages.

### 4.3 “Kinfluence” and Association Dynamics

Overall, our findings suggest that “kinfluence” plays a subtle role in shaping association patterns among adult bull sharks visiting the Shark Reef Marine Reserve. At the community level, we found weak support for individual bull sharks preferentially associating with relatives, and this faint signal appears to be driven by related females associating with one another. Although only one estimator produced a statistically significant relationship between relatedness and association strength, all three estimators recovered weak positive correlations in the same direction. Notably, the estimator that detected this relationship also exhibited the lowest variance in simulation (see Table S2), suggesting that the significant result may reflect greater precision rather than selective interpretation of a single estimator. The consistency in the direction and magnitude of the Mantel correlation coefficients across estimators suggests a subtle relationship between relatedness and association patterns may exist, albeit one that is relatively weak within this aggregation. This avenue of inquiry appears to warrant further investigation following a more comprehensive genetic sampling of the aggregation.

When categorical kinship was examined using a weighted kinship matrix, full sibling and half‐sibling dyads showed slightly stronger association preferences than non‐kin dyads. The detection of this effect despite the presence of only six identified full sibling dyads and 31 identified half‐sibling dyads suggests that these few close kin pairs may form stronger social connections in comparison to the non‐kin dyads which make up the majority of the population. Detecting kin‐based assortment under such conditions is inherently difficult, particularly in natural populations where close kin are rare, and relatedness values are generally low. Accordingly, the small Mantel correlation coefficients reported here indicate that kin exert a genuine, but only limited influence on association patterns within this aggregation. Individuals appear to associate largely independent of relatedness, implying that other drivers play a stronger role in structuring community‐level associations. Indeed, recent work on this aggregation has shown that sex and age‐class are positive predictors of network structure (Marosi et al. 2026b), as are personality traits (Marosi et al. 2026c).

Similar patterns have been observed in terrestrial vertebrate systems exhibiting fission‐fusion dynamics where kinship alone does not predict the formation or maintenance of social bonds (Best et al. 2014; Carter et al. 2013; Hirsch et al. 2013; Langergraber et al. 2007; Moscovice et al. 2017; Wilkinson et al. 2016). In such systems with consistent changes in group membership, kinship may operate alongside morphological and behavioral traits in addition to familiarity in shaping social preferences. The aggregation observed at the Shark Reef Marine Reserve may therefore represent a comparable context in which kinship does not solely determine social organization but makes contributions to it on multiple levels.

Across marine vertebrates, kinship can play a substantial role in structuring social associations, particularly in cetaceans, where relatedness strongly predicts grouping patterns and social cohesion (Frère et al. 2010; Foroughirad et al. 2019; Wiszniewski et al. 2010). Such systems, however, are typically characterized by prolonged parental care, complex social systems, and cohesion (Fox et al. 2017; Kasuya 1995; Mann 2009), features that differ markedly from the life history of most elasmobranchs. Nevertheless, kin‐mediated sociality has been reported in sharks and rays under some ecological conditions. For example, acoustic tracking of juvenile bluntnose sixgill sharks in Puget Sound revealed long‐term associations among full and half‐sibling littermates, with kin dyads exhibiting synchronized vertical movements in the water column (Andrews et al. 2009, 2010). Similarly, juvenile lemon sharks in the Bahamas showed evidence of kin‐biased associations during 1 year of a 3‐year study, although body size appeared to exert the strongest overall influence on assortment patterns (Guttridge et al. 2011). Together, these findings suggest that young sharks may forge social bonds at early life stages, particularly when they remain within restricted nursery habitats separated from sub‐adult and adult populations.

In contrast, studies of adult elasmobranchs have often found little or no evidence for kin‐structured social groups. Spotted eagle rays in Florida formed groups independently of relatedness, with group formation more likely related to predator defense (Ajemian et al. 2012; Newby et al. 2014), while adult blacktip reef sharks in Moorea also associated independently of relatedness (Mourier and Planes 2021). Our findings align more closely with these latter studies and suggest that although there may be subtle traces of kinship within our association structure, it does not broadly organize the adult aggregation. This likely reflects the fact that any kin‐biased associations that may be forged earlier in life become diluted by dispersal, mortality, and the mixing of individuals from multiple lineages within the adult population.

### 4.4 “Kinfluence” and Social Interaction Dynamics

In contrast to the weak signal observed in association structure, our fine scale analysis suggests that genetic relatedness exerts a stronger influence over social interaction dynamics. Most notably, relatedness was positively correlated with male–male interaction strength, indicating that when males do engage in same‐sex social interactions, these interactions are disproportionately directed towards kin. This is particularly interesting in light of work on the same population showing that males preferentially socially interact with females over other males (Marosi et al. 2026b). This suggests that the rare occasions on which males selectively interact with other males may be especially informative and may be influenced by familial relatedness.

Whether this preference is rooted in kin bonds or familiarity, and whether these bonds formed early in life or later though remains unknown. Within this aggregation, females are both physically larger and more numerous, while the population operates within an established hierarchy (Marosi et al. 2026d). Therefore, kin‐based male social interactions may reflect social bonds which have formed for the purpose of conflict amelioration, cooperation, or to conform with social hierarchies (Myrberg Jr and Gruber 1974; Whitehead 2002). Indeed, males who build strong social bonds with male conspecifics have been shown to sire more offspring, achieve higher social rank, and be more successful at forming coalitions (Gilby et al. 2013; McDonald 2007; Schülke et al. 2010).

The lack of corresponding signal when categorical kinship is used to examine social interaction strength does not necessarily contradict the relatedness‐based result we see for social interactions. Rather, it highlights an important distinction between methodological approaches. The COLONY framework assigns only close pedigree relationships such as full siblings and half siblings, while grouping more distant relationships, including third order relationships (first cousin) and lower into the non‐kin category. In our dataset, COLONY identified only two male–male half‐sibling dyads and no male–male full sibling dyads. Continuous relatedness estimators, by contrast, capture a broader gradient of genetic similarity. Thus, the Mantel test result could reflect stronger interaction strengths which exist among male–male fourth order dyads (second cousin, *r* ≥ 0.03125) and closer. Our use of these combined methodological approaches supports the interpretation that “kinfluence” in this population is subtle, context dependent, and not restricted solely to close kin.

### 4.5 Life History, Kin Recognition and Constraints on Kinfluence

The subtle “kinfluence” observed within the Shark Reef aggregation may reflect the life history and population structure of bull sharks in Fiji. Bull sharks within this study system belong to an insular population distributed throughout the Fijian archipelago (Devloo‐Delva et al. 2023; Glaus et al. 2020) and utilize restricted riverine and estuarine habitats during early life stages, where neonates, YOY, and juveniles occupy relatively confined nursery environments (Compagno et al. 2005; Cardeñosa et al. 2017; Glaus 2019; Simpfendorfer and Heupel 2011). Such spatial restriction likely increases repeated encounters among siblings and other related individuals during these formative years. In addition, evidence of parturition philopatry in Fiji further suggests maternal lineages repeatedly recruit to the same river systems (Brunnschweiler et al. 2024), contributing to the weak lineage structure detected in the present study.

Under these conditions kin or familiar recognition and social bonds established among related individuals early in life may persist into adulthood, even within a largely unrelated adult aggregation. The sensory and cognitive capabilities of bull sharks suggest that such recognition is possible. Bull sharks possess well‐developed olfactory, visual and electro sensory systems (Scheiber, Collin and Hart 2012; Whitehead 2002; Yopak et al. 2015) which may facilitate discrimination among conspecifics through stable sensory cues, where information about familiar or related individuals is retained over time as demonstrated in fishes (e.g., Gerlach et al. 2008; Gerlach and Wullimann 2021; Hain and Neff 2007). Kin recognition may arise through familiarity and prior association during shared occupancy of nursery habitats where related individuals co‐occur during early developmental stages (Alexander 1974; Gottlieb 1981; Gubernick 1981; Mateo 2004). Alternatively, phenotypic matching mechanisms, whereby individuals form recognition templates based on familiar conspecifics, may also contribute to discrimination of kin from non‐kin later in life (Holmes and Sherman 1983; Sherman et al. 1997; Tang‐Martinez 2001).

Several factors nevertheless operate against the development or persistence of strong kin structuring in this system. Bull sharks in Fiji experience substantial juvenile mortality. Although gravid females may produce litters of up to 13 pups (Bass et al. 1973; Compagno et al. 2005; Ebert et al. 2013), fisheries‐independent surveys of nursery areas on Viti Levu show rapid declines in juvenile capture rates within months of parturition, indicating substantial mortality at early life stages (Cardeñosa et al. 2017; Glaus, et al. 2019a). Illegal gillnetting in riverine and estuarine habitats, combined with natural predation pressure, drastically reduces the number of siblings that survive beyond the first years of life (Glaus, et al. 2019a; Marie et al. 2017; Paris et al. 2019; Vierus et al. 2018). Consequently, relatively few close kin are likely to survive long enough to recruit into the adult aggregation, which is consistent with the low frequency of elevated relatedness in our genetic dataset.

There are also methodological constraints; identifying kinship in elasmobranchs remains challenging because these species are often wide‐ranging, occur at low densities, and are difficult to sample comprehensively. In addition, relatedness estimates can be sensitive to marker choice, filtering strategy, and sample size, and there is still no unified analytical framework for relatedness estimation in sharks and rays (Amini et al. 2025). In our study, the genetic dataset represented only a subset of the total aggregation, and behavioral comparisons were limited to individuals who could be reliably identified underwater. Expanding genetic sampling and long‐term identification efforts would improve the power to detect kin relationships and evaluate their effects more comprehensively. Even so, the fact that subtle kin signals were detectable despite these limitations strengthens the interpretation that “kinfluence,” while characterized as weak at this juncture, is genuinely present in this system.

### 4.6 Provisioning and Opportunities for Social Behavior

Provisioned eco‐tourism sites increase opportunities for repeated encounters among individuals by attracting sharks to predictable food sources (Brunnschweiler and Barnett 2013). At the Shark Reef Marine Reserve, provisioning facilitates repeated spatio‐temporal overlap among conspecifics, creating conditions for the development of familiarity and social relationships. Similarly, studies conducted on captive small‐spotted cat sharks and lemon sharks have demonstrated that familiarity can drive association patterns (Jacoby et al. 2012; Keller et al. 2011). However, the provisioning itself does not dictate the observed social structure. Previous analyses conducted within this aggregation have revealed active social preferences, preferred and avoided relationships, and selective social interactions (Marosi et al. 2026b).

Provisioning appears to create the ecological context in which multiple factors, including sex, age, size, familiarity, personality (Marosi et al. 2026b; Marosi et al. 2026c), and to a limited extent, kinship may contribute to social dynamics. This ability to repeatedly observe identified individuals over many years provides a rare opportunity to investigate fine‐scale social structure in a wild elasmobranch population and may explain why subtle “kinfluence” patterns were detectable despite low overall relatedness and incomplete sampling of the aggregation.

## 5 Conclusions

This study provides the first direct test of whether genetic relatedness and categorical kinship influence the social structure and interaction dynamics of adult bull sharks. Although the aggregation visiting the Shark Reef Marine Reserve is not kin‐structured at the population level, our results reveal subtle evidence of “kinfluence.” Continuous relatedness measures revealed potentially weak positive relationships with both association and social interaction patterns, while the strongest signal emerged in male–male social interaction dynamics. Pedigree reconstruction further identified rare full‐sibling dyads that exhibited stronger associations when present. Notably, these signals were detectable even though the genetic dataset represents only a subset of the aggregation, suggesting that additional kin relationships may remain unsampled in the population.

These findings taken together suggest that “kinfluence” may operate in varied ways, but that its effects are subtle and occur alongside other important drivers of sociality, including sex, age, size, familiarity and personality traits (Marosi et al. 2026b; Marosi et al. 2026c). The patterns observed here are likely shaped by the life history of Fiji's bull sharks, in which viviparity and prolonged juvenile residency within riverine nursery habitats create opportunities for early kin encounters, while mortality and dispersal reduce the persistence of close kin into adulthood.

Future research focused on bull shark pups within the riverine nursery habitats will be critical for determining whether kin‐based social bonds form during early life and persist through juvenile development, into adulthood. Protecting these nursery environments will therefore be important not only for bull shark conservation in Fiji, but also for understanding the mechanisms underpinning social behavior in this species. By integrating long term behavioral observations with genetic relatedness and pedigree reconstruction, this study provides rare empirical support for kin‐mediated processes in a large marine predator. Our findings suggest that while kinship does not broadly organize the population, it nevertheless influences selective associations and social interactions. Importantly, the subtle nature of these effects is likely consistent with the life history of bull sharks, where juvenile mortality, dispersal and ontogenetic shifts progressively dilute kin structure into adulthood. Our findings therefore support the expanding view that elasmobranch social structures are shaped by multiple drivers including morphological and behavioral traits, familiarity and now kinship. More broadly, this study highlights the value of integrating behavioral and genomic approaches to facilitate our understanding of social organization in wide‐ranging, non‐model marine predators, where kin‐mediated processes may be difficult to detect, yet are still biologically meaningful.

## Author Contributions

**Natasha D. Marosi:** conceptualization (lead), data curation (lead), formal analysis (lead), funding acquisition (lead), investigation (lead), methodology (equal), visualization (equal), writing – original draft (lead), writing – review and editing (lead). **Darren P. Croft:** conceptualization (supporting), supervision (supporting), writing – review and editing (equal). **David M. P. Jacoby:** conceptualization (supporting), supervision (supporting), writing – review and editing (equal). **Samuel Ellis:** methodology (supporting), writing – review and editing (supporting). **Andrew M. Griffiths:** conceptualization (lead), methodology (lead), supervision (lead), writing – review and editing (equal).

## Funding

This work was supported by Hai Stiftung Shark Foundation; The Rufford Foundation Grant no. 35197‐1; Waitt Foundation; and Viti Leucas Society D.B.A. Fiji Shark Lab.

## Ethics Statement

This study was conducted with the knowledge and approval of the Ministry of Fisheries and Forests, Fiji and the traditional owners of Shark Reef. Ethics review and approval for this study was granted by the University of Exeter, Exeter, UK, FHLS Psychology Ethics Committee (No. 492960). All shark samples were shipped in accordance with the relevant import and exportation permits issued by the Ministry of Fisheries and Forests in Fiji and obtained by Diversity Arrays in Canberra, Australia.

## Conflicts of Interest

The authors declare no conflicts of interest.

## Supporting information


**Table S1:** Root Mean Squared Error (RMSE) between observed and expected values for pairs of simulated individuals of known relatedness (i.e., full‐sibling = 0.5; half‐sibling = 0.25, parent‐offspring = 0.5; first‐cousin = 0.125; second‐cousin = 0.03125 and unrelated = 0).
**Table S2:** Variance of observed values for pairs of simulated individuals of known relatedness (i.e., full‐sibling = 0.5; half‐sibling = 0.25, parent‐offspring = 0.5; first‐cousin = 0.125; second‐cousin = 0.03125 and unrelated = 0).
**Table S3:** Three chosen estimators used in analysis of genotyped individuals and their respective Root Mean Squared Errors (and variance) between observed and expected values for pairs of simulated individuals of known relatedness (i.e., full sibling = 0.5; parent‐offspring = 0.5; half‐sibling = 0.25; first cousins, aunt/uncle‐niece/nephew = 0.125; second cousins 0.0325).

**Table S4:** Pedigree‐based kinship assignments inferred using COLONY (posterior probability > 0.80) under the relaxed SNP filtering strategy. Dyads are characterized as full siblings (FS) or half siblings (HS) according to COLONY output. First order kin (FS) include dyads potentially representing either full sibling or parent‐offspring relationships as COLONY does not reliably distinguish these without known parents. Age‐class, sex, size (body length) and first observation date are provided for biological context. All dyads shown represent the complete set of inferred close‐kin relationships within the genotyped sample. Dates follow format (D/M/Y).

## Data Availability

Data are available on Figshare: https://figshare.com/s/3f0568ecf8c4e991465a.
