# Supplementary material for: Is Blood Thicker Than Water? The Influence of Kin Within a Bull Shark Social Network
Source: Ecol Evol. 2026 Aug 5;16(8):e74050. doi: 10.1002/ece3.74050 (PMC13442586; doi:10.1002/ece3.74050)
Supplement: Supplementary file 1 [file ECE3-16-e74050-s001.docx]

| **Method** | **Parent-**  **offspring** | **Full-sibs** | **Half-sibs** | **First Cousin** | **Second Cousin** | **Unrelated** | **Mean** |
| --- | --- | --- | --- | --- | --- | --- | --- |
| Triadic | 0.018522 | 0.017200 | 0.021713 | 0.009073 | 0.016879 | 0.011885 | 0.015879 |
| Milligan | 0.018517 | 0.017264 | 0.021771 | 0.009223 | 0.017025 | 0.011943 | 0.015957 |
| EMIBD9 | 0.018205 | 0.016903 | 0.021036 | 0.007737 | 0.015535 | 0.019303 | 0.016453 |
| L & R | 0.021265 | 0.021928 | 0.026553 | 0.020010 | 0.018341 | 0.014982 | 0.020513 |
| Q & G | 0.024306 | 0.021086 | 0.024043 | 0.016408 | 0.023895 | 0.024448 | 0.022364 |
| GRM | 0.020985 | 0.025907 | 0.024416 | 0.023885 | 0.021025 | 0.020406 | 0.022771 |
| Wang_U | 0.025071 | 0.019300 | 0.022876 | 0.014860 | 0.027162 | 0.027479 | 0.022791 |
| L & L | 0.025118 | 0.019596 | 0.023001 | 0.014309 | 0.027180 | 0.027601 | 0.022801 |
| Wang | 0.025255 | 0.020026 | 0.023234 | 0.013969 | 0.027516 | 0.027903 | 0.022984 |
| GCTA | 0.022061 | 0.031324 | 0.029155 | 0.035142 | 0.018492 | 0.014980 | 0.025192 |
| Ritland | 0.022358 | 0.031204 | 0.029705 | 0.035276 | 0.018486 | 0.014932 | 0.025327 |
| EMIBD9_1 | 0.025525 | 0.029984 | 0.033964 | 0.023997 | 0.019069 | 0.021014 | 0.025592 |

**Table S1 |** Root Mean Squared Error (RMSE) between observed and expected values for pairs of simulated individuals of known relatedness (i.e. full-sibling = 0.5; half-sibling = 0.25, parent-offspring = 0.5; first-cousin = 0.125; second-cousin = 0.03125 and unrelated = 0).

| **Method** | **Parent-**  **offspring** | **Full-sibs** | **Half-sibs** | **First Cousin** | **Second Cousin** | **Unrelated** | **Mean** |
| --- | --- | --- | --- | --- | --- | --- | --- |
| EMIBD9 | 0.000330 | 0.000288 | 0.000439 | 0.000060 | 0.000213 | 0.000111 | 0.000240 |
| Triadic | 0.000345 | 0.000298 | 0.000460 | 0.000060 | 0.000285 | 0.000090 | 0.000256 |
| Milligan | 0.000344 | 0.000297 | 0.000461 | 0.000062 | 0.000291 | 0.000091 | 0.000258 |
| EMIBD9_1 | 0.000418 | 0.000374 | 0.000533 | 0.000179 | 0.000307 | 0.000135 | 0.000324 |
| L & R | 0.000457 | 0.000468 | 0.000698 | 0.000400 | 0.000335 | 0.000226 | 0.000431 |
| Q & G | 0.000595 | 0.000448 | 0.000576 | 0.000266 | 0.000564 | 0.000598 | 0.000508 |
| GRM | 0.000445 | 0.000677 | 0.000601 | 0.000576 | 0.000432 | 0.000420 | 0.000525 |
| Wang_U | 0.000627 | 0.000375 | 0.000514 | 0.000219 | 0.000735 | 0.000756 | 0.000538 |
| L & L | 0.000627 | 0.000387 | 0.000522 | 0.000203 | 0.000736 | 0.000762 | 0.000539 |
| Wng | 0.000638 | 0.000405 | 0.000540 | 0.000195 | 0.000745 | 0.000783 | 0.000551 |
| GCTA | 0.000491 | 0.000989 | 0.000857 | 0.001247 | 0.000334 | 0.000225 | 0.000690 |
| Ritland | 0.000505 | 0.000962 | 0.000881 | 0.001249 | 0.000340 | 0.000225 | 0.000694 |

**Table S2 |** Variance of observed values for pairs of simulated individuals of known relatedness (i.e. full-sibling = 0.5; half-sibling = 0.25, parent-offspring = 0.5; first-cousin = 0.125; second-cousin = 0.03125 and unrelated = 0).

The twelve relatedness methods that were assessed are:

1. Queller & Goodnight (1989); coded in figures and table as “Q & G”
2. Li et al. (1993); coded as “L & L”
3. Ritland (1996); coded as “Ritland”
4. Lynch & Ritland (1999) coded as “L & R”
5. Wang (2002); coded as “Wang”
6. Wang (2017); coded as “Wang_U”
7. Genetic relationship matrix (Lee et al., 2011) coded as “GCTA”
8. Additive relationship matrix (Endelman & Jannink, 2012) coded as “GRM”
9. Milligan (2003); coded as “Milligan”

10. Wang (2007); coded as “Triadic”

11. Wang (2022) without taking into account inbreeding to estimate relatedness; coded as “EMIBD9”

12. Wang (2022) with taking into account inbreeding to estimate relatedness; coded as “EMIBD9_1

| **Method** | **Parent-Offspring** | **Full-Sibs** | **Half-Sibs** | **First Cousin** | **Second Cousin** | **Unrelated** | **Mean** |
| --- | --- | --- | --- | --- | --- | --- | --- |
| Wang 2007 | 0.018522  (0.000345) | 0.017200  (0.000298) | 0.021713  (0.000460) | 0.009073  (0.000060) | 0.016879  (0.000285) | 0.011885  (0.000090) | 0.015879  (0.000256) |
| Milligan | 0.018517  (0.000344) | 0.017264  (0.000297) | 0.021771  (0.000461) | 0.009223  (0.000062) | 0.017025  (0.000291) | 0.011943  (0.000091) | 0.015957  (0.000258) |
| Wang 2022 | 0.018205  (0.000330) | 0.016903  (0.000288) | 0.021036  (0.000439) | 0.007737  (0.000060) | 0.016879  (0.000213) | 0.019303  (0.000111) | 0.016453  (0.000240) |

**Table S3 |** Three chosen estimators used in analysis of genotyped individuals and their respective Root Mean Squared Errors (and variance) between observed and expected values for pairs of simulated individuals of known relatedness (*i.e.* full sibling = 0.5; parent-offspring = 0.5; half-sibling = 0.25; first cousins, aunt/uncle-niece/nephew = 0.125; second cousins 0.0325).
