# Supplementary material for: Is Blood Thicker Than Water? The Influence of Kin Within a Bull Shark Social Network
Source: Ecol Evol. 2026 Aug 5;16(8):e74050. doi: 10.1002/ece3.74050 (PMC13442586; doi:10.1002/ece3.74050)
Supplement: Supplementary file 2 [file ECE3-16-e74050-s002.docx]

| Dyad ID | Individual A,  Individual B | Kin Category | Posterior probability | Sex A,  Sex B | Age-Class A,  Age-Class B | Size A (cm),  Size B (cm) | Date first obs A*,  Date first obs B* |
| --- | --- | --- | --- | --- | --- | --- | --- |
| 1 | BF111, BF014 | FS | 1.00 | F, F | Adult, Adv -Adult | 302, 319 | 18/07/2022, 01/02/2009 |
| 2 | BF111, BF058 | FS | 1.00 | F, F | Adult, Adult | 302, 299 | 18/07/2022, 01/08/2018 |
| 3 | BF069, BU005 | FS | 1.00 | F, UKN | Adult, Adult | 289, 299 | 11/10/2019, 20/02/2012 |
| 4 | BF014, NA | FS | 1.00 | F, F | Adv-Adult, Adult | 319, 299 | 01/02/2009, 01/08/2018 |
| 5 | BM024, BF163 | FS | 1.00 | M, F | Adult, Adult | 250, N/A | 01/08/2022, 24/11/2016 |
| 6 | BF124, BF026 | FS | 1.00 | F, F | Adult, Adult | 278, 305 | 16/09/2022, 01/11/2009 |
| 7 | BM013, BF003 | FS | 1.00 | M, F | Adult, Adv-Adult | 260, 317 | 01/02/2018, 01/07/2003 |
| 8 | BM018, BF026 | FS | 1.00 | M, F | Adult, Adult | 262, 305 | 01/04/2019, 01/11/2009 |
| 9 | BM027, BF117 | FS | 1.00 | M, F | Adult, Adult | 263, 260 | 18/07/2022, 15/08/2022 |
| 10 | BF002, BF097 | FS | 1.00 | F, F | Adv-Adult, Adult | 325, 273 | 01/06/2003, 01/04/2023 |
| 11 | BF019, BF156 | FS | 1.00 | F, F | Adult, Adult | 284, N/A | 01/06/2009, 17/07/2017 |
| 12 | BF111, BM019 | HS | 1.00 | F, M | Adult, Sub-Adult | 302, 228 | 18/07/2022,21/01/2020 |
| 13 | BF069, BM022 | HS | 1.00 | F, M | Adult, Adult | 289, 246 | 11/10/2019, 01/08/2022 |
| 14 | BF069, BF103 | HS | 1.00 | F, F | Adult, Adult | 289, 288 | 11/10/2019, 01/07/2011 |
| 15 | BF014, BF084 | HS | 1.00 | F, F | Adv-Adult, Sub-Adult | 319, 241 | 01/02/2009, 01/06/2022 |
| 16 | BM024, NA | HS | 0.966 | M, UKN | Adult, Adult | 250, N/A | 01/08/2022, 13/03/2012 |
| 17 | BM001, BF093 | HS | 1.00 | M, F | Adv-Adult, Adult | 268, 310 | 01/06/2003, 01/01/2023 |
| 18 | BF031, BF083 | HS | 1.00 | F, F | Adult, Adv-Adult | 297, 317 | 01/05/2011, 01/05/2022 |
| 19 | BF018, BM014 | HS | 1.00 | F, M | Adult, Adult | 328, 283 | 01/03/2009, 06/02/2018 |
| 20 | BF093, BM029 | HS | 1.00 | F, M | Adult, Adult | 310, 258 | 01/01/2023, 01/06/2022 |
| 21 | BF106, BM007 | HS | 1.00 | F, M | Adult, Adult | 278, 270 | 04/08/2022, 01/03/2010 |
| 22 | BF056, BF041 | HS | 1.00 | F, F | Adult, Adult | 298, 283 | 02/07/2022, 01/12/2013 |
| 23 | BF058, BM019 | HS | 1.00 | F, M | Adult, Sub-Adult | 299, 228 | 01/08/2018, 21/01/2020 |
| 24 | BF058, BF084 | HS | 1.00 | F, F | Adult, Sub-Adult | 299, 241 | 01/08/2018, 01/06/2022 |
| 25 | BM025, BF020 | HS | 1.00 | M, F | Adult, Adult | 252, 299 | 02/02/2023, 01/06/2009 |
| 26 | BM025, BF068 | HS | 1.00 | M, F | Adult, Adult | 252, 308 | 02/02/2023, 19/09/2019 |
| 27 | BM009, BM005 | HS | 1.00 | M, M | Adult, Adv-Adult | 254, 277 | 01/02/2011, 01/06/2009 |
| 28 | BF009, BM006 | HS | 1.00 | F, M | Adult, Adult | 301, 276 | 10/02/2007, 01/02/2010 |
| 29 | BF010, BF077 | HS | 0.942 | F, F | Adult, Sub-Adult | 268, 244 | 01/10/2007, 31/01/2022 |
| 30 | BM022, BM013 | HS | 1.00 | M, M | Adult, Adult | 246, 259 | 01/08/2022, 01/02/2018 |
| 32 | BM022, BF103 | HS | 1.00 | M, F | Adult, Adult | 246, 288 | 01/08/2022, 01/07/2011 |
| 32 | BM003, BF085 | HS | 1.00 | M, F | Adult, Adult | 244, 237 | 01/03/2008, 01/07/2022 |
| 33 | BF124, BM008 | HS | 1.00 | F, M | Adult, Adult | 278, 270 | 16/09/2022, 01/08/2018 |
| 34 | BF028, BF087 | HS | 0.889 | F, F | Adult, Adult | 287, 248 | 01/01/2010,01/08/2022 |
| 35 | BF028, BF053 | HS | 1.00 | F, F | Adult, Adult | 287, 289 | 01/01/2010, 01/12/2016 |
| 36 | BF038, BF042 | HS | 1.00 | F, F | Adult, Adult | 280, 285 | 21/06/2013, 01/12/2013 |
| 37 | BF036, BF021 | HS | 1.00 | F, F | Adult, Adult | 311, 301 | 01/06/2012, 01/06/2009 |
| 38 | BF020, BF068 | HS | 1.00 | F, F | Adult, Adult | 299, 308 | 01/06/2009, 19/09/2019 |
| 39 | BF037, BF008 | HS | 1.00 | F, F | Adult, Adult | 291, 298 | 01/06/2013, 01/01/2007 |
| 40 | BF131, BF027 | HS | 1.00 | F, F | Adult, Adv-Adult | 271, 319 | 08/11/2022, 01/11/2009 |
| 41 | BF131, BU007 | HS | 1.00 | F, UKN | Adult, Adult | 271, N/A | 08/11/2022, 28/06/2012 |
| 42 | BF129, BF045 | HS | 1.00 | F, F | Adult, Adult | 292, 288 | 31/10/2022, 01/06/2014 |
| 43 | BF129, BU006 | HS | 1.00 | F, UKN | Adult, Adult | 292, N/A | 31/10/2022, 19/07/2012 |
| 44 | NA, BF039 | HS | 1.00 | F, F | Adult, Adult | 278, 286 | 17/09/2022, 01/08/2013 |
| 45 | BF085, BF021 | HS | 1.00 | F, F | Sub-Adult, Adult | 237, 301 | 01/07/2022, 01/06/2009 |
| 46 | BM018, BM008 | HS | 1.00 | M, M | Adult, Adult | 262, 270 | 01/04/2019, 01/08/2018 |
| 47 | BM012, BF033 | HS | 1.00 | M, F | Adult, Adult | 264, 286 | 01/09/2017, 01/07/2011 |
| 48 | NA, BU007 | HS | 1.00 | F, UKN | Adult, Adult | 283, N/A | 01/11/2023, 28/06/2012 |
| 49 | BM027, BF015 | HS | 1.00 | M, F | Adult, Adult | 263, 304 | 18/07/2022, 01/02/2009 |
| 50 | BF117, BF015 | HS | 1.00 | F, F | Adult, Adult | 260, 304 | 15/08/2022, 01/02/2009 |
| 51 | BM006, BF158 | HS | 1.00 | M, F | Adult, Adult | 276, N/A | 01/02/2010, 02/10/2017 |
| 52 | BF019, BF160 | HS | 0.999 | F, F | Adult, Adult | 284, N/A | 01/06/2009, 10/10/2016 |
| 53 | BF103, BU005 | HS | 1.00 | F, UKN | Adult, Adult | 288, N/A | 01/07/2011, 20/02/2012 |
| 54 | BU001, BM035 | HS | 1.00 | UKN, M | Adult, Adult | N/A, N/A | 00/00/2012, 29/01/2017 |
| 55 | BU004, BF039 | HS | 1.00 | UKN, F | Adult, Adult | N/A, 286 | 13/03/2012, 01/08/2013 |
| 56 | BU004, BF163 | HS | 1.00 | UKN, F | Adult, Adult | N/A, N/A | 13/03/2012, 24/11/2016 |
| 57 | BF039, BF163 | HS | 1.00 | F, F | Adult, Adult | 286, N/A | 01/08/2013, 24/11/2016 |

**Table S4.** Pedigree-based kinship assignments inferred using COLONY (posterior probability > 0.80) under the relaxed SNP filtering strategy. Dyads are characterized as full siblings (FS) or half siblings (HS) according to COLONY output. First order kin (FS) include dyads potentially representing either full sibling or parent-offspring relationships as COLONY does not reliably distinguish these without known parents. Age class, sex, size (body length) and first observation date are provided for biological context. All dyads shown represent the complete set of inferred close-kin relationships within the genotyped sample. Dates follow format (D/M/Y).

*****for tagged or unknown individuals this date corresponds to date of genetic tissue sampling.
